# Risk factors for poorer respiratory outcomes in adolescents and young adults born preterm

**DOI:** 10.1136/thorax-2022-219634

**Published:** 2023-05-19

**Authors:** Elizabeth F Smith, Naomi R Hemy, Graham L Hall, Andrew C Wilson, Conor P Murray, Shannon J Simpson

**Affiliations:** 1 Wal-Yan Respiratory Research Centre, Telethon Kids Institute, Perth, Western Australia, Australia; 2 School of Allied Health, Curtin University, Perth, Western Australia, Australia; 3 Respiratory and Sleep Medicine, Perth Children's Hospital, Perth, Western Australia, Australia; 4 Medical Imaging, Perth Children's Hospital, Perth, Western Australia, Australia

**Keywords:** Imaging/CT MRI etc, Lung Physiology, Paediatric Lung Disaese

## Abstract

**Rationale:**

The respiratory outcomes for adult survivors of preterm birth in the postsurfactant era are wide-ranging with prognostic factors, especially those encountered after the neonatal period, poorly understood.

**Objectives:**

To obtain comprehensive ‘peak’ lung health data from survivors of very preterm birth and identify neonatal and life-course risk factors for poorer respiratory outcomes in adulthood.

**Methods:**

127 participants born ≤32 weeks gestation (64%, n=81 with bronchopulmonary dysplasia (BPD), initially recruited according to a 2 with-BPD:1 without-BPD strategy), and 41 term-born controls completed a lung health assessment at 16–23 years, including lung function, imaging and symptom review. Risk factors assessed against poor lung health included neonatal treatments, respiratory hospitalisation in childhood, atopy and tobacco smoke exposure.

**Measurements and main results:**

Young adults born prematurely had greater airflow obstruction, gas trapping and ventilation inhomogeneity, in addition to abnormalities in gas transfer and respiratory mechanics, compared with term. Beyond lung function, we observed greater structural abnormalities, respiratory symptoms and inhaled medication use. A previous respiratory admission was associated with airway obstruction; mean forced expiratory volume in 1 s/forced vital capacity z-score was −0.561 lower after neonatal confounders were accounted for (95% CI −0.998 to –0.125; p=0.012). Similarly, respiratory symptom burden was increased in the preterm group with a respiratory admission, as was peribronchial thickening (6% vs 23%, p=0.010) and bronchodilator responsiveness (17% vs 35%, p=0.025). Atopy, maternal asthma and tobacco smoke exposure did not influence lung function or structure at 16–23 years in our preterm cohort.

**Conclusions:**

Even after accounting for the neonatal course, a respiratory admission during childhood remained significantly associated with reduced peak lung function in the preterm-born cohort, with the largest difference seen in those with BPD. A respiratory admission during childhood should, therefore, be considered a risk factor for long-term respiratory morbidity in those born preterm, especially for individuals with BPD.

**WHAT IS ALREADY KNOWN ON THIS TOPIC**

**WHAT THIS STUDY ADDS**

- Our study reports that a spectrum of impairment exists for young adults born ≤32 weeks gestation, from normal to severe. We show that, for those with BPD, a respiratory admission in childhood is a significant risk factor for reduced lung function in adolescence and young adulthood, while maternal asthma and personal history of atopy had no discernible effect.

**HOW THIS STUDY MIGHT AFFECT RESEARCH, PRACTICE OR POLICY**

- Our findings reveal that a childhood history of respiratory hospital admission should be a key consideration in the management of preterm children and adults. As the number of survivors of preterm birth continues to grow due to increases in the rate of preterm births, and advances in neonatal care, identifying those most at risk is key evidence needed to generate clinical guidelines for the management of these patients.

## Introduction

 The lungs undergo extensive growth and development throughout childhood to a ‘peak’ function in young adulthood. This peak is followed by a natural decline with increasing age.^1^ Failure to reach an appropriate peak in lung function during young adulthood is a significant predictor of all-cause mortality^2^ and a major risk factor for chronic obstructive pulmonary disease (COPD), accounting for up to half of all cases in adults.^3^ Early-life respiratory infections, environmental exposures or a childhood asthma diagnosis are key contributors to a failure to reach potential peak lung function, and potentially a faster natural decline in lung function.^4^

The Early Life Origins of (respiratory) Disease pose some unique concerns for the 11% of the global population born preterm (<37 weeks gestation).^5^ Life-saving treatments, such as mechanical ventilation or supplemental oxygen, can elicit injury on the incompletely developed lung of the preterm infant. Other insults, such as early-life respiratory infections, also disproportionally affect preterm infants, likely due to an immature innate immune system and disruption to normal immune development.^6 7^ In children with bronchopulmonary dysplasia (BPD), antenatal^8^ and personal^9^ smoke exposure are associated with lung function decline, while secondhand smoke exposure is associated with increased hospital admissions.^10^ Modifiable postnatal factors are likely to contribute to decreased peak lung function in a cumulative way, and increase respiratory morbidity and mortality throughout adult life.^11^ Avoiding a ‘second hit’ to the respiratory system has been postulated to be central to mitigating adult respiratory disease following preterm birth.^12^

Several studies have established that survivors of preterm birth have altered lung structure, increased respiratory symptoms and low lung function (although largely by spirometry only) throughout life^13^,^16^ and that these deficits are exacerbated by diagnosis of chronic lung disease of infancy, bronchopulmonary dysplasia (BPD).^17^,^19^ Further, it has been shown by our group (in the same cohort) and others, that lung function trajectories move further from predicted throughout childhood^8^ and adolescence.^9^ However, identifying risk factors, especially those encountered following the intensive neonatal period, for poor long-term respiratory outcomes have received little focus.

Comprehensive assessments of lung structure, function and symptoms profiles near ‘peak’ are of particular value, and are rare in modern survivors of very preterm birth.^20 21^ This study aimed to provide a comprehensive cross-section of lung health in very preterm born adolescents and young adults (near ‘peak’), and importantly, assess the role of antenatal, neonatal and early childhood exposures on peak lung function, lung structure and respiratory symptoms. We hypothesised that preterm birth would be associated with reduced lung function and ongoing respiratory morbidity in young adulthood and that this would be worse for those with early-life risk factors, including increased respiratory support, respiratory hospitalisation and exposure to tobacco smoke.

## Methods

### Participants

Adolescents and young adults from the pre-existing Western Australian Lung Health in Prematurity cohort^8 13 16^ were invited to attend a research appointment aged 16–23 years. Detailed information on the cohort has been previously published.^13^ Briefly, the preterm group were born at King Edward Memorial Hospital ≤32 weeks gestation between 1997 and 2003. BPD was defined as at least 28 days supplemental oxygen requirement as assessed at 36 weeks postmenstrual age.^22^ In the cohort, there is a deliberate recruitment strategy of 1 term: 1 preterm (without BPD): 2 preterm (with BPD). The incidence of BPD in infants born at 32 weeks’ gestation or earlier during these birth years is 28% in Western Australia.^8^ Therefore, the proportion of those with of BPD in this cohort (67%) is over double that expected in the general population born 32 weeks gestation or less. Due to this recruitment strategy, the overall preterm cohort is born at an earlier gestation and lower birth weight than would be expected in the general population.^16^

We have previously shown the subgroups with and without BPD are representative of the corresponding populations in the local area, born during these birth years.^13^ Briefly, in these subgroups, there is no difference in birth weight or days of supplemental oxygen compared with the corresponding eligible populations. While gestational age (GA) is not different for preterm participants with BPD, those without BPD had a slightly lower GA than the non-recruited cohort.^13^

Term controls were recruited at earlier cohort follow-ups,^13 16^ briefly they were born at ≥37 weeks gestation, with no history of cardiopulmonary disease or recurrent respiratory symptoms at time of recruitment. Written informed consent and assent (where appropriate) was obtained from the participant (and their guardian where appropriate).

### Assessment of lung function

Assessment of lung function included spirometry, diffusing capacity for the lung, whole body plethysmography, multiple breath washout, fractional exhaled nitric oxide and oscillometry carried out according to American Thoracic Society (ATS) and European Respiratory Society (ERS) guidelines. The bronchodilator response was assessed by spirometry following the administration of 400 µg salbutamol via spacer. Where possible, lung function outcomes were expressed as z-scores (additional detail on lung function methodology is provided in online supplemental data).

### Assessments of lung structure

CT images of the chest were acquired during inspiration and expiration in both term-born and preterm-born participants. Inspiratory and expiratory volumetric images spanned from the lung apex to the diaphragm at ultra-low radiation dose (dose length product: 8 mGy×cm, regardless of age) (Somatom Definition Force, Siemens, Erlangen). The chest CT images were consensus scored using a modification of the scoring system described by Aukland *et al*.^13 23^ Extent scores are directly related to the number of affected lobes (maximum=6) except for collapse/consolidation (maximum=2) with a possible total CT score of 50. Scorers were blinded to all participant information. A random subset of CT scans (n=22) were rescored within 6 months, by the same observers. Cronbach’s alpha and the intraclass correlation coefficient (ICC) estimate with 95% CIs were calculated using IBM SPSS Statistics for Windows, V.27.0, based on a single-rating, absolute-agreement, 2-way mixed-effects model.

### Assessment of risk factors for poor lung health

A history of household tobacco smoke exposure, respiratory hospitalisation, maternal asthma, personal asthma, eczema or hay fever were assessed via a questionnaire, with a positive history at any of the cohort follow-ups included in the analysis.^24^ Additionally, respiratory symptoms in the 3 months prior to the visit was assessed using the same questionnaire. Neonatal data extracted from medical records included GA, birth anthropometrics, antenatal and neonatal steroid exposure, confirmed sepsis during neonatal intensive care unit (NICU) admission, number of surfactant doses, days of supplemental oxygen, mechanical ventilation and continuous positive airway pressure (CPAP).

### Statistical analysis

Data were analysed using IBM SPSS Statistics for Windows, V.27.0. Data were assessed for normality using the Shapiro-Wilk test and reported as means and SD for normally distributed data and medians and IQR for non-normally distributed data. Differences between groups were assessed by paired T-test, Mann-Whitney U test, one-way analysis of variance or the Kruskal Wallis test, as appropriate. For normally distributed data, Bonferroni correction was applied to account for possible type 1 errors due to multiple testing; for non-parametric data, pairwise comparisons were used. χ² analysis was used for differences in proportion between groups.

The association between predictors (neonatal treatments, respiratory admissions, CT scores) and lung function was investigated using univariate and multivariable linear regression models. Multivariable regression models were built using all independent variables, which were entered into the model individually and the significance of the change in R^2^ at each step was analysed. The effect size was defined as the unstandardised β coefficient. Normal distribution of residuals was confirmed by visualisation of histogram and Q-Q plots. To evaluate the association between neonatal factors and respiratory admissions with young adult CT scores, univariate and multivariable negative binomial regression was performed. Dichotomous outcomes with dichotomous predictors are analysed using logistic regression and ORs are reported.

## Results

### Study participants at follow-up visit

A total of 168 participants (41 term; 127 preterm) were included (online supplemental figure E1) at a mean (SD) age of 19.31 (1.39). Of the preterm participants, 19 had mild BPD while 62 had moderate-to-severe BPD.^22^ Participant demographics are shown in table 1. Recent respiratory symptoms, respiratory-based hospitalisations, exposure to tobacco smoke and atopy were all higher in the preterm born population (table 1). Asthma diagnosis (ever), asthma medication use and exercise-induced wheeze in the past 3 months were increased in both the no-BPD and BPD groups compared with term-born controls. Recent cough, rattle and shortness of breath were increased in the BPD group only (table 1).

**Table 1:** Participant demographics and risk factors at adolescent/young adult follow-up visit

	Term	Preterm	PretermNo BPD	BPD
Participants, n	41	127	46	81
Sex				
Females, n (%)	19 (46)	56 (44)	17 (37)	39 (48)
Males, n (%)	22 (54)	71 (56)	29 (63)	42 (52)
Age (years)	19.3 (1.5)	19.3 (1.4)	19.0 (1.2)	19.5 (1.4)
Height (cm)	175.4 (9.6)	169.0 (9.7)*	171.5 (9.4)	167.5 (9.5)*
Weight (kg)	70.2 (14.7)	65.9 (17.6)	70.6 (22.3)	63.2 (13.6)
BMI	22.7 (3.9)	22.8 (4.5)	23.6 (5.2)	22.4 (4.0)
SpO_2_ (%)	98 (1)	98 (1)	98 (1)	98 (1)
Heart rate (bpm)	76 (10)	79 (12)	75 (11)	**81 (12)^†^**
Systolic blood pressure (mm Hg)	125 (10)	126 (12)	129 (12)	125 (12)
Diastolic blood pressure (mm Hg)	72 (8)	71 (9)	71 (10)	71 (9)
Symptoms in the 3 months prior to test visit, n (%)				
Asthma medication	0 (0)	21 (16.5)*	8 (17.4)*	13 (16.0)*
Wheeze	1 (2.4)	15 (11.8)	5 (10.9)	10 (12.3)
Wheeze during exercise	2 (4.9)	23 (18.1)*	9 (19.6)*	14 (17.3)*
Cough	20 (48.8)	64 (50.4)	17 (37.0)	**47(58.0)** †
Rattle	2 (4.9)	21 (16.5)	2 (4.3)	**19 (23.5)** * †
Shortness of breath	5 (12.2)	37 (29.1)*	12 (26.1)	25 (30.9)*
Reported respiratory hospitalisations (lifetime)				
Respiratory hospitalisation, n (%)	3 (7.3)	63 (49.6)*	15 (32.6)*	**48 (59.3)** * †
No of respiratory hospitalisations, median (IQR)	0 (0–0)	0 (0–1)*	0 (0–1)	1 (0–1)*
No of respiratory hospitalisations, range	0–2	0–12	0–10	0–12
Smoking, n (%)				
Ever smoker	2 (4.9)	6 (4.7)	3 (6.5)	3 (3.7)
Household smoke exposure	8 (19.5)	59 (46.5)*	23 (50.0)*	36 (44.4)*
Asthma/atopy*,* n (%)				
Asthma ever	5 (12.2)	67 (52.8)*	29 (63.0)*	38 (46.9)*
Hay fever ever	10 (24.4)	58 (45.7)*	22 (47.8)*	36 (44.4)*
Eczema ever	15 (36.6)	44 (34.6)	17 (37.0)	27 (33.3)
Atopy (hay fever or eczema ever)	17 (41.5)	77 (60.6)*	29 (63.0)*	48 (59.3)
Physician diagnosed maternal asthma	4 (9.8)	29 (22.8)	12 (26.1)*	17 (21.0)

Participant demographics at the time of testing are presented as mean (SD) unless otherwise indicated. The ‘preterm’ group is composed of all preterm participants, recruited using a deliberate 1 preterm without BPD: 2 preterm with BPD strategy.

Bold font indicates statistical significance.

* p<0.05 compared with term-born group.

† p<0.05 compared with no-BPD group.

BMIbody mass indexBPDbronchopulmonary dysplasiaSpO2peripheral oxygen saturation

We did not observe evidence of selection bias when we compared data from participants that did and did not take part in this adolescent/young adult follow-up. The preterm participants returning for this follow-up were born at a median (IQR) gestation of 27.0 (25.0–29.4) weeks, comparable to the wider cohort (p=0.255). Other neonatal information describing the preterm cohort are presented in table 2. We report no difference in neonatal course (eg, proportion preterm with BPD, 45.7% vs 41.5%, p=0.866) or lung function (eg, forced expiratory volume in 1 s (FEV_1_) at 11 years; mean difference −0.03 z-scores, 95% CI −0.47 to 0.41, p=0.878) between preterm-born participants who did, and did not attend on this occasion.

**Table 2:** Neonatal information for preterm participants at adolescent/young adult follow-up

	Preterm	Preterm	
		No BPD	BPD
Participants, n	127	46	81
Gestational age (PMA)	27.0 (25.0, 29.4)	29.9 (28.4, 30.5)	25.7 (24.9, 27.0)*
Birth weight (g)	930 (750, 1195)	1323 (1165, 1574)	825 (685, 935)*
Birth weight z-score (mean, SD)	−0.16 (0.89)	−0.12 (0.83)	−0.19 (0.93)
Duration of oxygen supplementation (days)	57 (3, 95)	1 (0, 3.3)	91 (62.5, 104)*
Duration of mechanical ventilation (days)	5 (0.5, 28)	0 (0, 1.2)	20 (5, 36.5)*
Duration of CPAP (days)	6 (1, 19.6)	1 (0, 3.6)	14 (5.1, 24.8)*
Surfactant administered, n (%)	94 (74.0)	22 (47.8)	72 (88.9)
Antenatal steroids, n (%)	100 (78.7)	35 (76.1)	65 (80.2)
Postnatal steroids, n (%)	35 (27.6)	4 (8.7)	31 (38.3)

Continuous neonatal variables are presented as median (IQR), except birth weight z-score (mean, SD). Birthweight z-score was calculated from Fenton growth charts for preterm infants.^45^ Antenatal steroids includes those who received the intervention a minimum of 24 hours prior to delivery only. The ‘preterm’ group is composed of all preterm participants, recruited using a deliberate 1 preterm without BPD: 2 preterm with BPD strategy.

Bold font indicates statistical significance.

* Represents significant difference between the preterm groups with and without BPD (p<0.05).

BPDbronchopulmonary dysplasiaCPAPcontinuous positive airway pressurePMApostmenstrual age

### Lung function in adolescents and young adults born preterm

#### Forced flows and volumes

No difference in forced vital capacity (FVC) was observed, however, airflow obstruction was evidenced by reduced spirometric values (FEV_1_, FEV_1_/FVC, forced expiratory flow (FEF)_25-75_, FEF_75_) in young adults born preterm, with increased severity in those with BPD (table 3). Abnormally low lung function (defined as FEV_1_, FEV_1_/FVC, FEF_25–75_ or FEF_75_ ≤−1.64 z-scores) was observed in 6 of the term controls (15.8%), 15 (35.7%) of the preterm group without BPD, and 39 (54.2%) of those with BPD (p<0.001). Baseline FEV_1_ was less than −1.64 z-scores in 2 of the term controls (5.0%), 6 (14.0%) of the preterm group without BPD and 24 (30.8%) of those with BPD (p=0.002). The corresponding values for FEV_1_/FVC<−1.64 z-scores were: term controls (n=4, 10.5%), preterm group without BPD (n=13, 31.0%) and preterm with BPD (n=30, 41.7%, p=0.004).

**Table 3:** Lung function in term and very preterm-born adolescents and young adults

	Term controls (reference)	Preterm	Preterm	
			No-BPD	BPD
Forced flows and volumes (spirometry)				
n successful	40	121	43	78
FEV_1_ z-score	−0.03 (1.14)	**−0.88(1.16)** *	−0.48 (1.10)	**−1.10(1.14)** * †
FEV1 z-score post-BD	0.34 (1.12)	**−0.28(1.02)** *	0.00 (1.07)	**−0.43(0.97)** *
n successful	38	113	41	72
FVC z-score	0.09 (1.07)	−0.01 (1.02)	0.21 (1.03)	−0.14 (1.01)
FEV_1_/FVC z-score	−0.19 (1.08)	**−1.17(1.12)** *	**−0.99(1.14)** *	**−1.27(1.10)** *
FEF_25-75_ z-score	−0.27 (1.11)	**−1.29(1.15)** *	**−0.98(1.15)** *	**−1.47(1.13)** *
Bronchodilator assessment, n	39	119	42	77
No with BDR	3 (7.7%)	**31(26.1%)** *	7 (16.7%)	**24(31.2%)** *
Gas exchange (DLCO)				
n successful	41	120	45	75
DLCO z-score	1.48 (0.97)	**0.85(1.08)** *	1.06 (1.11)	**0.73(1.05)** *
VA z-score	0.74 (0.88)	0.64 (1.18)	0.71 (1.15)	0.60 (1.20)
KCO z-score	0.89 (0.85)	**0.33(1.01)** *	0.49 (1.07)	**0.24(0.97)** *
Lung volumes (whole body plethysmography)				
n successful	40	123	44	79
TLC z-score	0.27 (0.70)	0.28 (0.82)	0.34 (0.81)	0.25 (0.83)
FRC z-score	0.42 (0.69)	0.55 (0.92)	0.42 (0.89)	0.62 (0.93)
RV z-score	0.47 (0.53)	0.69 (0.79)	0.51 (0.74)	**0.79 (0.81)** *
RV/TLC (%) z-score	0.50 (0.73)	0.77 (0.93)	0.52 (0.85)	**0.91 (0.95)** *
Respiratory system mechanics (oscillometry)				
n successful	41	125	45	80
Rrs_5_ z-score	1.25 (0.87)	1.33 (1.18)	1.20 (1.46)	1.40 (0.98)
Fres z-score	1.13 (1.04)	**1.90(1.42)** *	1.42 (1.42)	**2.17(1.36)** * †
AX z-score	1.25 (0.85)	**1.92(1.15)** *	1.61 (1.23)	**2.10(1.08)** * †
Xrs_5_ z-score	0.75 (0.92)	**1.36(1.49)** *	1.04 (1.41)	**1.54(1.51)** *
Rrs_5-20_ (cmH_2_O.s/L)	0.10 (0.34)	**0.51(0.81)** *	0.40 (0.77)	**0.57(0.83)** *
Median (IQR)				
Airway Inflammation				
n successful	41	125	45	80
FeNO (ppm)	19 (12, 25)	15 (10, 26)	19 (12, 32)	**13 (10, 19)** †
Ventilation distribution (multiple breath wash-out)				
n successful	36	97	40	**57** *
LCI	6.54 (5.99, 7.19)	**7.26(6.46, 8.19)** *	7.14 (5.96, 7.79)	**7.39(6.63, 8.49)** *
MR1 (M1/M0)	1.79 (1.50, 1.97)	**1.94(1.57, 2.19)** *	1.94 (1.52, 2.15)	**1.94(1.61, 2.34)** *
MR2 (M2/M0)	5.68 (4.16, 6.98)	**6.92(4.59, 8.85)** *	6.90 (4.14, 8.57)	**7.02(4.70, 10.11)** *
Airway resistance (plethysmography)				
n successful	40	119	43	76
sGaw (1 /s*cmH_2_0)	0.20 (0.17, 0.25)	**0.15(0.11, 0.23)** *	0.17 (0.11, 0.25)	**0.15(0.11, 0.20)** *

Data are presented as mean (SD) unless otherwise indicated, except for the number of successful tests. The ‘preterm’ group is composed of all preterm participants, recruited using a deliberate 1 preterm without BPD: 2 preterm with BPD strategy.

Bold font indicates statistical significance.

* p<0.05 compared with term-born group.

† p<0.05 compared with no-BPD group.

AXarea under the reactance curveBDbronchodilatorBDRbronchodilator responseBPDbronchopulmonary dysplasiaDLCOdiffusing capacity of the lung for carbon monoxideFEF_25–75_forced expiratory flow at 25%–75% of the pulmonary volumeFeNOfractional exhaled nitric oxideFEV_1_forced expiratory volume in 1 sFRCfunctional residual capacityFresresonant frequencyFVCforced vital capacityKCOcarbon monoxide transfer coefficientLCILung Clearance IndexMR1/2moment ratio 1/2Rrs_5-20_difference in respiratory system resistance between 20 and 5 HzRVresidual volumesGawspecific airway conductanceTLCtotal lung capacityVAalveolar volumeXrs_5_respiratory system reactance at 5 Hz

#### Bronchodilator responsiveness

Of the 29 young adults born preterm with a baseline FEV_1_<−1.64 z-scores (and acceptable pre- and post-bronchodilator spirometry), 19 (65.5%) had a significant bronchodilator response, while the remaining 10 (34.5%) did not. A bronchodilator response was observed in 7.7% of term controls, 16.7% of those preterm without BPD, increasing to 31.2% in those with BPD (p=0.010).

#### Gas exchange

Gas exchange (diffusing capacity of the lung for carbon monoxide (DLCO)) was reduced in the preterm cohort compared with term-born controls, and worst in those with BPD, with a mean DLCO z-score difference of −0.63 (95% CI −0.25 to −1.01) and KCO z-score difference of −0.55 (95% CI −0.21 to −0.90). There was no observed difference in alveolar volume in those born ≤32 weeks GA (table 3).

#### Static lung volumes by whole body plethysmography

Young adults with BPD had an increased residual volume (RV) and RV as a percentage of total lung capacity (RV/TLC) compared with term-born controls, suggestive of gas trapping (table 3). There were no differences observed in other measures of static lung volumes, including TLC and functional residual capacity in those born preterm (table 3).

#### Ventilation homogeneity by multiple breath washout

There was increased ventilation inhomogeneity in those born preterm compared with term-born controls, evidenced by increased Lung Clearance Index and the first and second moment ratios of the gas washout (M1/M0 and M2/M0, respectively). The greatest increase in ventilation inhomogeneity was observed in the BPD group (table 3).

#### Airway inflammation

Fractional exhaled nitric oxide was lower in the preterm group with BPD, compared with term-born controls (p=0.03) (table 3).

#### Respiratory system mechanics (by plethysmography and oscillometry)

Young adults born preterm had poorer peripheral lung mechanics than term-born controls, which was worst in those with BPD, evidenced by decreased respiratory system reactance at 5 Hz (Xrs_5_), area under the reactance curve (AX) and resonant frequency (Fres) z-scores (table 3). Respiratory resistance z-scores measured by oscillometry were not significantly increased in those born preterm. However, the plethysmographic measure of airway resistance that is independent of lung volume (sGaw) revealed decreased airway conductance in those born ≤32 weeks GA, which was further decreased in those with BPD (table 3).

### Lung structure in preterm adolescents and young adults

Structural abnormalities were present in 88% of adolescents and young adults born ≤32 weeks gestation, increasing to 92% in the BPD group (p=0.047). Of note, structural abnormalities were also present in 61% of the term-born control group, however, were more extensive in those born 32 weeks gestation or less. In those with BPD, structural abnormalities were more extensive; total scores ranged from 0 to 16 in those without BPD, increasing to 0–22 in those with BPD, out of a maximum score of 50^13 23^ (see table 4 for median and IQR).

**Table 4:** The presence and extent of chest CT abnormalities in adolescents and young adults born very preterm

	Term(n=38)	Preterm(n=125)	Preterm without BPD(n=46)	Preterm with BPD(n=79)
Linear/triangular subpleural opacities				
Presence, n participants (%)	16 (42%)	**84(67%)** *	22 (48%)	**62(79%)** * †
Extent (CT score) (IQR)	0 (0–1)	2 (0–4)*	0 (0–2)	**2 (1–4)** * †
Decreased pulmonary attenuation—inspiration				
Presence	0 (0%)	**14(11%)** *	2 (4%)	12 (15%)*
Extent	0 (0–0)	**0(0–0)** *	0 (0–0)	**0(0–0)** * †
Decreased pulmonary attenuation—expiration				
Presence	13 (34%)	86 (69%)*	26 (57%)*	**60(76%)** * †
Extent	0 (0–1)	2 (0–4)*	1 (0–4)*	**2 (1 – 4)** * †
Decreased bronchial: arterial ratio				
Presence	0 (0%)	3 (2%)	2 (4%)	1 (1%)
Extent	0 (0–0)	0 (0–0)	0 (0–0)	0 (0–0)
Bronchiectasis				
Presence	2 (5%)	5 (4%)	3 (7%)	2 (3%)
Extent	0 (0–0)	0 (0–0)	0 (0–0)	0 (0–0)
Bronchial wall thickening				
Presence	0 (0%)	**18(14%)** *	4 (9%)	**14(18%)** *
Extent	0 (0–0)	**0(0–0)** *	0 (0–0)	**0(0–0)** *
Bullae				
Presence	1 (3%)	0 (0%)	0 (0%)	0 (0%)
Extent	0 (0–0)	0 (0–0)	0 (0–0)	0 (0–0)
Emphysema				
Presence	0 (0%)	4 (3%)	0 (0%)	4 (5%)
Extent	0 (0–0)	0 (0–0)	0 (0–0)	0 (0–0)
Collapse/consolidation				
Presence	1 (3%)	9 (7%)	3 (7%)	6 (8%)
Extent	0 (0–0)	0 (0–0)	0 (0–0)	0 (0–0)
Structural abnormalities on chest CT				
Presence, n participants (%)	23 (61%)	110 (88%)*	37 (80%)*	**73(92%)** * †
Total CT score	1 (0–2)	4 (2–8)*	3 (1–6)*	5 (3–9)*†

Presence indicates the number (%) of preterm young adults with the structural abnormality described. Extent scores were derived according to a modification of the scoring matrix by Aukland *et al*^23^ where scores are directly related to the number of affected lobes (maximum=6) except for collapse/consolidation (maximum=2) with a possible total CT score of 50. Extent scores are expressed as median (IQR). The ‘preterm’ group is composed of all preterm participants, recruited using a deliberate 1 preterm without BPD: 2 preterm with BPD strategy.

Bold font indicates statistical significance.

* p<0.05 compared with term-born group.

† p<0.05 compared with no-BPD group.

BPDbronchopulmonary dysplasia

Linear and triangular pleural opacities and elicitation and/or an exaggeration of decreased pulmonary attenuation during expiration were the most commonly observed abnormalities (observed in 37% and 69% of the preterm cohort, respectively), and were more prevalent and severe in those with BPD. Bronchial wall thickening and decreased pulmonary attenuation during inspiration were also increased in those born very preterm (table 4).

The intraobserver reliability of CT scorers was excellent with an absolute agreement ICC of 0.89 (95% CI 0.69 to 0.96, p<0.001) and Cronbach’s alpha of 0.95.

### Association between lung structure, function and symptoms in preterm-born adolescents and young adults

**Figure 1:**
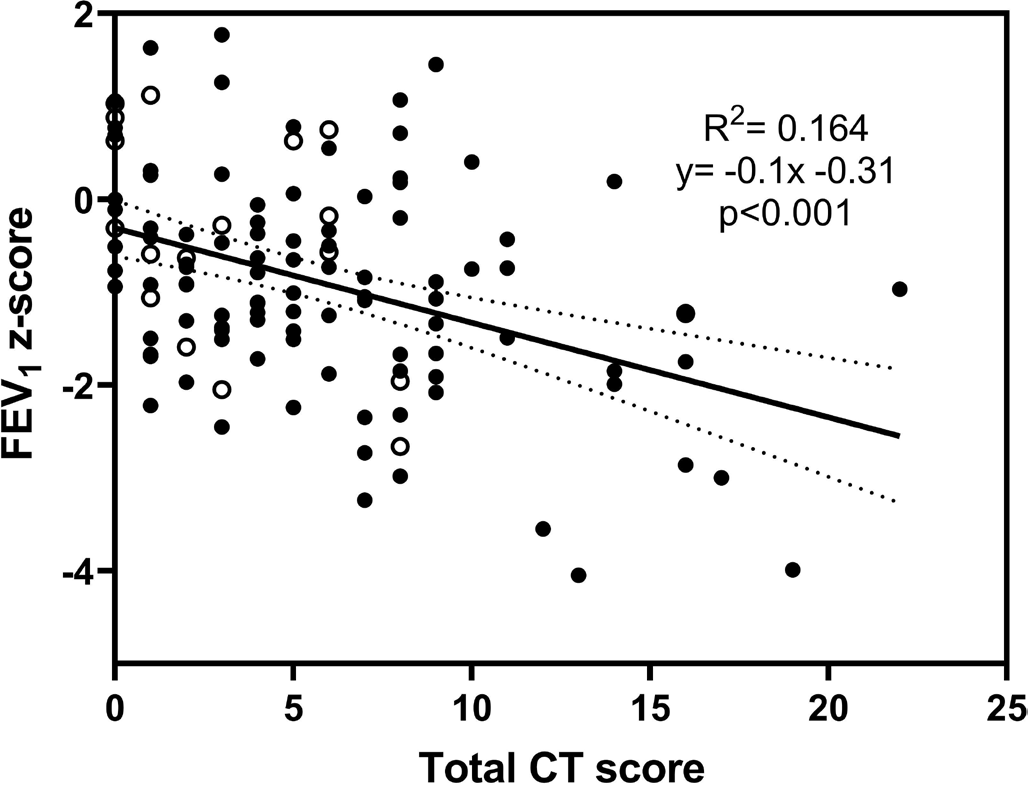
Lung structure abnormalities are related to lung function in preterm adolescents and young adults. FEV_1_ z-score is plotted against total CT score for preterm adolescents and young adults with (closed-circle) and without (open-circle) bronchopulmonary dysplasia. Linear regression analysis is displayed, with 95% CIs (dotted lines). FEV_1_, forced expiratory volume in 1 s.

Airway obstruction was observed in preterm individuals with more extensive structural abnormalities (online supplemental table E1); for example, FEV_1_ decreased −0.1 z-scores for every 1 unit increase in the total CT score (95% CI −0.06 to −0.14). This association was weak (R^2^=0.164) although highly statistically significant (p<0.001, figure 1). Similarly, adolescents and young adults born very preterm with wheeze during exercise (n=23, 18.1%), had increased airflow obstruction; the mean difference in FEV_1_/FVC ratio was 0.544 z-scores (95% CI 0.076 to 0.101, p=0.024). Additional associations between individual lung function measures and structural abnormalities are presented in online supplemental tables E1–E9.

### Neonatal factors associated with impaired lung function and structure in adolescence and young adulthood

Univariate analyses relating neonatal factors (including gestation, birth weight z-score, days of supplemental O_2_, mechanical ventilation, CPAP, surfactant doses, antenatal steroids, postnatal steroids, etc) to each lung function or structure outcome are presented in online supplemental tables E10–E17. Univariate analysis revealed multiple associations between neonatal factors and adolescent/young adult lung function, however, the associations were attenuated when confounders were adjusted for. This is with the exception of increased RV and RV/TLC in those who had received longer durations of mechanical ventilation, with an effect size of 0.13 z-score change in RV/TLC for every additional 1 week of mechanical ventilation (β=0.019, 95% CI 0.003 to 0.035, p=0.021, online supplemental table E12). Negative binomial multivariable regression analysis also showed that increased requirement for supplemental O_2_, mechanical ventilation or postnatal steroids, as well as a respiratory admission following discharge from the NICU, were risks for a higher peribronchial thickening score during adolescence or young adulthood (online supplemental table E17).

### Lifetime exposures are associated with impaired respiratory function, structure and symptoms in adolescents and young adults

#### Smoking

**Figure 2:**
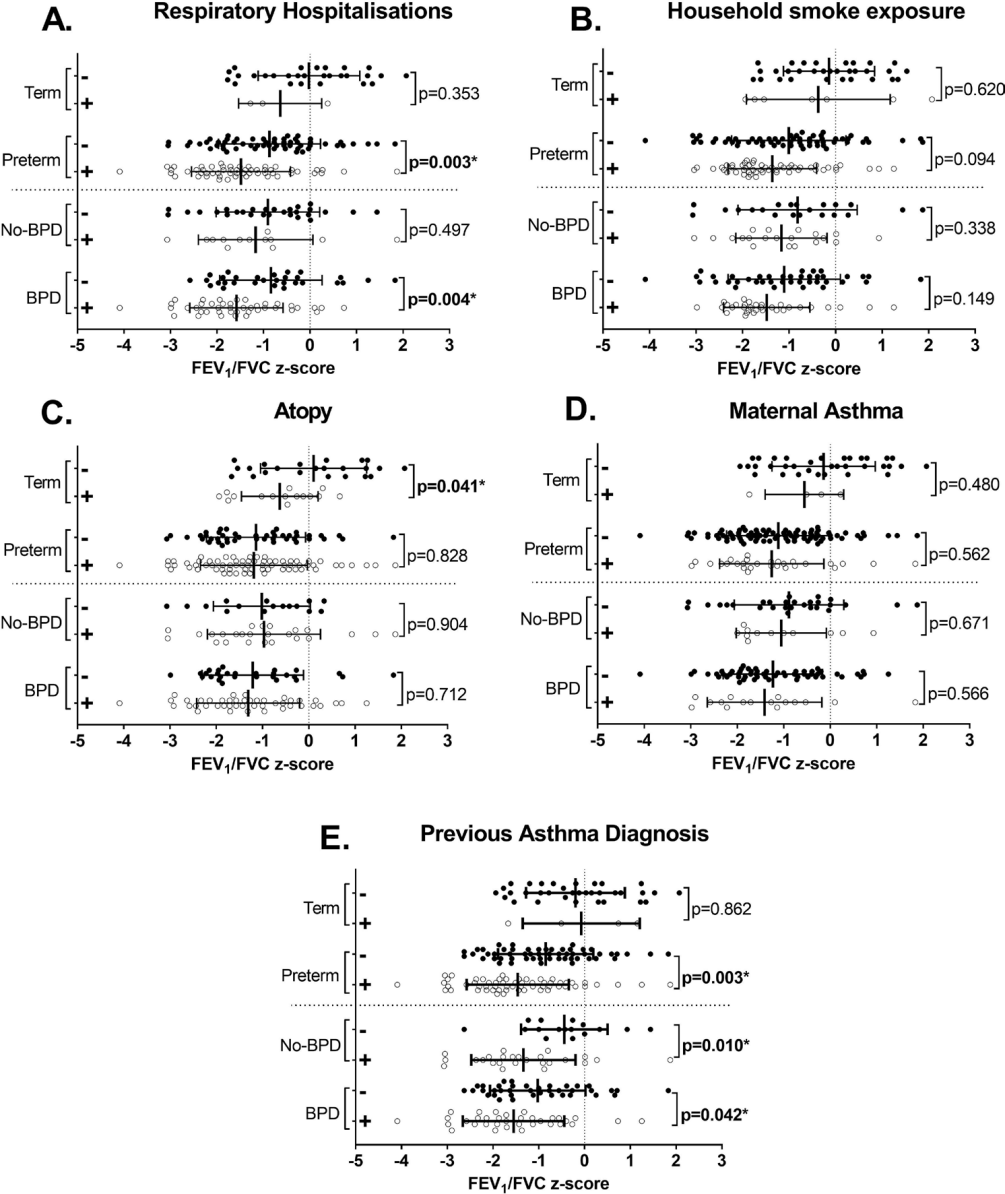
Risk factors influence lung function at age 19. The association between (**A**) respiratory hospitalisations, (**B**) household smoke exposure, (**C**) atopy, (**D**) maternal asthma and (**E**) a previous asthma diagnosis, and airway obstruction (FEV_1_/FVC z-score) in young adults born preterm (no-BPD and BPD), and term-born controls. Error bars represent mean±SD. *Represents significant difference between the with (+, open circle)) and without (−, closed circle) (factor) for each group. BPD, bronchopulmonary dysplasia; FEV_1_, forced expiratory volume in 1 s; FVC, forced vital capacity.

The number of ‘ever smokers’ was not different between the term and preterm groups (p=0.968), with less than 5% of both groups reporting a history of smoking (table 1). Individuals born preterm who reported personal or household exposure to tobacco smoke did not have reduced lung function or increased structural lung damage compared with those not reporting tobacco smoke exposure (eg, figure 2).

The reporting of respiratory symptoms at rest was increased in those who had experienced household exposure to tobacco smoke (46% vs 73%, p=0.002), primarily attributable to the increased prevalence of cough (41% vs 61%, p=0.026).

#### Respiratory admissions are associated with respiratory structure, function and symptoms in adolescents and young adults

There was a 10-fold greater risk of a respiratory admission during childhood in those born very preterm, compared with term (OR 10.172, 95% CI 2.958 to 34.977, p<0.001). Respiratory admissions were overwhelmingly reported in childhood; all except one respiratory admission was recorded at the 9–12 years follow-up or earlier.^13^ Rates of respiratory admission to hospital, were 32.6% and 59.3% in those without and with BPD, respectively (p<0.001). Additionally, those with BPD had more respiratory admissions per person (p=0.021); 22.2% (n=18) had 3 or more admissions, compared with 13.0% (n=6) in the preterm group without BPD.

In those born preterm, spirometry outcomes were reduced in those with a previous respiratory admission. Mean FEV_1_/FVC was 0.61 z-scores lower in those born preterm with a respiratory admission (95% CI 0.21 to 1.02, p=0.003) and this difference was greatest in those with BPD (−0.74 z-scores, 95% CI −0.24 to –1.24, p=0.004) (figure 2). When adjusted for collinearities between respiratory admissions and neonatal factors, admissions remained significant with an effect size of −0.561 z-scores (95% CI −0.998 to –0.125, p=0.012, online supplemental table E10). A positive bronchodilator response was observed in 35.0% of those with a respiratory admission, compared with 16.9% without (p=0.025).

The presence of peribronchial thickening on chest CT was increased in those who reported a previous respiratory hospitalisation (6% vs 23%, p=0.010), as was the incidence of those reporting daytime wheeze (5% vs 18%, p=0.022), cough (36% vs 59%, p=0.010) or ‘rattle’ (5% vs 24%, p=0.002) in the past 3 months.

### Asthma and atopy associations with lung function and structure in adolescence and young adulthood

#### Participant eczema/hay fever

Term-born participants with a history of atopy (eczema or hay fever ever) (n=17, 41.5%), had a reduced FEV_1_/FVC z-score (mean difference −0.729, 95% CI −0.032 to −1.425, p=0.041). No difference in FEV_1_/FVC was observed in those born preterm with (n=77, 60.6%) and without atopy (mean difference 0.047, 95% CI −0.382 to 0.477, p=0.948). No other lung function, structure or symptoms outcomes were different between atopic participants and non-atopic participants.

#### Previous asthma diagnosis

A previous respiratory admission was a risk factor for an asthma diagnosis (OR 3.12, 95% CI 1.51 to 6.45); 66.6% (n=42) of those reporting a previous respiratory admission had received an asthma diagnosis, compared with 39.1% (n=25) of those who had not. Those born preterm with a previous asthma diagnosis had a greater degree of airflow obstruction (FEV_1_/FVC mean difference −0.609 z-scores, 95% CI −0.206 to –1.013), a greater extent of bronchiectasis (p=0.032) and peribronchial thickening (p=0.017) and increased prevalence of wheeze, both at rest (3% vs 19%, p=0.005) and with exertion (8% vs 27%, p=0.007).

#### Maternal asthma

There was no difference in any lung function outcomes, CT scores or respiratory symptoms in individuals with a maternal asthma history (eg, figure 2).

## Discussion

Here, we present a comprehensive assessment of lung function, symptoms and structure at close to peak function in contemporary survivors of very preterm birth, that is, following the routine introduction of exogenous surfactant in the NICU during the early 1990s. We demonstrated a significant burden of respiratory disease, including airflow limitation prebronchodilator and postbronchodilator, reduced gas transfer, ventilation inhomogeneity, increased symptoms and structural lung abnormalities, with the worst deficits observed in those with BPD. Importantly, we identify risk factors for lower than predicted peak lung function in this population, finding that early-life respiratory admissions were associated with reduced peak lung function and increased peribronchial thickening. A history of maternal asthma or atopy (hay fever or eczema ever) had no discernible effect on later lung function for those born ≤32 weeks gestation.

We report clinically relevant lung function impairment in adolescent and young adult survivors of very preterm birth, which is exacerbated in those with BPD. These findings extend previous studies.^25^,^28^ The existing literature in adolescent and young adult survivors of preterm birth has been difficult to interpret to date^29^ for reasons that include small sample size, a lack of suitable control groups and limited examples of comprehensive physiological testing. Further, previous studies have largely composed of adolescents and young adults who were born in the presurfactant era. Contemporary survivors with BPD are born at much earlier gestation and have a vastly different pulmonary pathophysiology compared with ‘old BPD’.^30^ This lack of clear evidence about the long-term impact of contemporary preterm birth has been cited as a key contributor to a clinician ‘blind spot’, where few adult respiratory physicians routinely consider early-life factors (ie, preterm birth) during patient assessment.^31^ Such suggestions are concerning as failure to achieve optimal peak lung function, and a vulnerability to accelerated decline, has raised concerns that those born early are at risk of developing a COPD-like phenotype in early adulthood.^32^ Indeed, studies suggest that those born prematurely have >2 fold increased risk of COPD in middle age^33^ and the mortality risk remains increased across the entire lifespan.^34^ Of significant note, 10% of our cohort had persistent airflow obstruction (FEV_1_/FVC<LLN), after the administration of a bronchodilator, satisfying the spirometric diagnostic criteria for COPD. This rate is twice that observed for COPD in Australia in those over 45 years of age.^35^

The potentially increased risk of COPD after preterm birth is especially pertinent for those exposed to tobacco smoke. Concerningly, in the preterm group we observed rates of household smoke exposure twice that of the control group, an observation likely linked to the interplay between low socioeconomic status, cigarette smoking and preterm birth.^36^ Urinary cotinine analysis was not performed to confirm smoking status, however, a personal history of smoking has previously been found to be reliable in this population.^37^ While we failed to observe a reduced FEV_1_ in those with tobacco smoke exposure (p=0.094, figure 2), we have previously noted in the same cohort that household tobacco smoke exposure was associated with increased declines in lung function throughout childhood.^8^ Similarly, Doyle *et al* observed negligible difference in lung function between smokers and non-smokers aged 18, however, crucially showed personal smoking is associated with lung function decline from 8 to 18 years.^9^ Continued follow-up of preterm born cohorts including smoking status will be essential to fully understand the implications of tobacco smoke exposure over time in this at-risk population.

We show that structural abnormalities are very common (88%) near peak lung health in survivors of contemporary preterm birth, the proportion and extent of which is increased in those with BPD. Consistent with the limited number of chest CT imaging studies,^23 38^ we report that linear and triangular opacities were the most commonly observed abnormality. However, a particular strength of this study is the inclusion of imaging healthy term-born controls, who also have high rates of linear and triangular opacities, raising the question about their importance as an ‘abnormality’. We report relatively low rates of emphysema in this population of adolescents and young adults with BPD (5%), compared with individuals of the same age, from the same centre born in the presurfactant era (47%).^38^ However, this difference may also be explained by potential recruitment bias in the previous study, with some subjects recruited from respiratory clinics.

In this study factors beyond the NICU were associated with suboptimal peak lung function for survivors of preterm birth. The European Respiratory Society recently published the first guidelines for the long-term clinical management of those with BPD.^39^ One of the eight critical questions identified pertained to the role of early-life infection (day care attendance), however, no articles were available to guide recommendations. Our data reiterate the importance of avoiding a ‘second hit’ to the respiratory system to improve adult outcomes.^12^ We show that a previous (infection related) respiratory admission was more impactful on lung health at age 19 years than neonatal factors, although we could not exclude that a respiratory admission after discharge could be a marker for pre-existing, premorbid pulmonary status. Notwithstanding, our findings raise hope for prophylactic therapeutic or life-style intervention, but also concern that those born ≤32 weeks gestation, especially those with BPD, are susceptible to further lung damage after leaving the NICU. Of concern, rehospitalisation rates for preterm babies with a respiratory virus are high (~40% of the preterm population, including ~65% of those with BPD),^40^ with similar rates of rehospitalisation in our cohort (59.3% in those with BPD). Early-life viral infections are associated with increased likelihood of later wheezing, asthma and reduced lung function in the term-born population,^4 41 42^ but the sparse studies after preterm birth have only focused on small numbers with RSV-related hospitalisations during infancy.^43 44^ Future prospective studies will be important to determine which respiratory viruses lead to hospitalisations and lower peak lung function for those born preterm.

### Limitations

Our data are limited by the parental reporting of hospitalisations, and therefore, our inability to identify the causative agent for the early-life respiratory admission. Further, we were unable to identify a precise age for each hospitalisation as the number of respiratory hospitalisations and an approximate age range was reported. A strength of this study was, however, that hospitalisation data were collected when participants were 5 years and 11 years of age, as well as at the current visit, ensuring a shorter time frame on recall. Regarding recall bias, we cannot exclude that those with current symptoms were not more likely to recall a previous respiratory admission. Similarly, confounding factors unaccounted for in this analysis could be related to both having a respiratory event during childhood and lower lung function in adult life. The greatest deficits in lung function following a respiratory admission were observed in those with BPD (figure 2). Our understanding of the associations between respiratory admission and later-life outcomes for the subgroup without BPD is limited due to the smaller size of this group (n=46 vs n=81) and the lower rate of respiratory hospitalisations (33% in those without-BPD vs 59% with-BPD). We were similarly limited in our ability to detect differences in other instances where the sample size was limited, for example, only three in the term-born control group reported a previous respiratory admission. Regardless, we provide important information on the potential role of early-life infections on lung health outcomes during adulthood.

The proportion of this cohort with BPD is higher than would be expected in a general population born 32 weeks gestation or less due to a deliberate recruitment strategy. It was anticipated that individuals with BPD would have the most complex and heterogeneous lung disease however, BPD (as defined once at 36 weeks corrected GA before the preterm infant has reached full term) is an imperfect predictor of long-term outcomes. We have, therefore, included both those with and without a BPD diagnosis in the analysis of risk factors for poorer outcomes. While those with and without BPD are representative of respective populations in the local area,^8^ the reader should be aware that lung function deficits as described in the whole preterm cohort may be more severe than could be expected in a general population born ≤32 weeks due to this recruitment strategy.

Lastly, due to the large volume of the data presented and the intention to identify risk factors for lower peak lung function, longitudinal analysis was out of the scope of this manuscript. Future longitudinal analyses will complement these findings.

## Conclusion

In conclusion, adolescence and young adulthood is supposed to be near the ‘peak’ of human lung health. However, survivors of preterm birth in the contemporary era commonly report respiratory symptoms, have structural lung damage and lower than expected lung function. As such, the evidence is mounting that many survivors are at increased risk of early-onset COPD and should be more closely monitored in the clinical setting and provided with anticipatory guidance on smoking, vaping and employment. Our study indicates that monitoring may be particularly important for those with an early-life readmission to hospital for a respiratory infection, however, it may not be until the fourth, fifth and sixth decades of life that the full impacts of early-life insults are fully realised.

## supplementary material

10.1136/thorax-2022-219634 online supplemental file 1

## Data Availability

Data are available on reasonable request.
