# Supplementary material for: Risk factors for poorer respiratory outcomes in adolescents and young adults born preterm
Source: Thorax. 2023 May 19;78(12):e219634. doi: 10.1136/thorax-2022-219634 (PMC11881051; doi:10.1136/thorax-2022-219634)
Supplement: online supplemental file 1 [file thorax-78-12-s001.pdf]

Title: Risk factors for respiratory morbidity in adolescents and young adults born preterm

Authors: Elizabeth F. Smith[1,2], Naomi R. Hemy[1], Graham L. Hall[1,2], Andrew C. Wilson[1,2,3], Conor P. Murray[3] and Shannon J. Simpson[1,2]

Online Data Supplement

Assessment of lung function (full details):

Assessment of lung function included spirometry, diffusing capacity for the lung, whole body plethysmography (Medisoft BodyBox 5500, Medisoft Corporation, Sorrines, Belgium); multiple breath washout (Exhalyzer D, Ecomedics, Duernten, Switzerland/EasyOne Pro Lab, NDD Medical Technologies, Zurich, Switzerland); fractional exhaled nitric oxide (HypAir FeNO, Medisoft Corporation, Sorrines, Belgium); and oscillometry (Tremoflo, Thorasys, Montreal, Canada). All lung function tests were performed according to ATS/ERS guidelines.[E1–6] The bronchodilator response was assessed by spirometry following the administration of 400mcg salbutamol via spacer, with a significant response defined as an improvement of ≥12% and 200ml in FEV_1 or FVC.[E7] Where possible, lung function outcomes were expressed as z-scores according to the Global Lung Function Initiative equations.[E8–10] Oscillometry outcomes were expressed as z-scores according to the reference equations published by Oostveen *et al*,[E11] with the exception of Rrs_{5-20}, expressed as an absolute difference. In adults, lung clearance index (LCI), moment ratio 1 and 2 (MR1, MR2) and specific airway conductance (sGaw), are independent of anthropometrics, and thus no adjustments have been made.[E12,13]

E1 Graham BL, Steenbruggen I, Miller MR, Barjaktarevic IZ, Cooper BG, Hall GL *et al.* Standardization of Spirometry 2019 Update. An Official American Thoracic Society and European Respiratory Society Technical Statement. *Am J Respir Crit Care Med* 2019; **200**: e70–e88.

E2 Graham BL, Brusasco V, Burgos F, Cooper BG, Jensen R, Kendrick A *et al.* 2017 ERS/ATS standards for single-breath carbon monoxide uptake in the lung. *Eur Respir J* 2017; **49**: 1–31.

E3 Wanger J, Clausen JL, Coates a., Pedersen OF, Brusasco V, Burgos F *et al.* Standardisation of the measurement of lung volumes. *Eur Respir J* 2005; **26**: 511–522.

E4 Robinson PD, Latzin P, Verbanck S, Hall GL, Horsley A, Gappa M *et al.* Consensus statement for inert gas washout measurement using multiple- and singlebreath tests. *Eur Respir J* 2013; **41**: 507–522.

E5 ATS/ERS. ATS/ERS recommendations for standardized procedures for the online and offline measurement of exhaled lower respiratory nitric oxide and nasal nitric oxide, 2005. *Am J Respir Crit Care Med* 2005; **171**: 912–930.

E6 King GG, Bates J, Berger KI, Calverley P, Melo L De, Dellacà RL *et al.* Technical standards for respiratory oscillometry. *Eur Respir J* 2020; **55**: 1–21.

E7 Pellegrino R, Viegi G, Brusasco V, Crapo RO, Burgos F, Casaburi R *et al.* Interpretative strategies for lung function tests. *Eur Respir J* 2005; **26**: 948–968.

E8 Quanjer PH, Cole TJ, Hall GL, Culver BH. Muti-ethnic reference values for spirometry for thee 3-95 years age range: the global lung function 2012 equations. *Eur Respir J* 2013; **40**: 1324–1343.

E9 Stanojevic S, Graham BL, Cooper BG, Thompson BR, Carter KW, Francis RW *et al.* Official ERS technical standards: Global Lung Function Initiative reference values for the carbon monoxide transfer factor for Caucasians. *Eur Respir J* 2017; **50**. doi:10.1183/13993003.00010-2017.

E10 Hall GL, Filipow N, Ruppel G, Okitika T, Thompson B, Kirkby J *et al.* Official ERS technical standard: Global lung function initiative reference values for static lung volumes in individuals of european ancestry. *Eur Respir J* 2021; **57**. doi:10.1183/13993003.00289-2020.

E11 Oostveen E, Boda K, Van Der Grinten CPM, James AL, Young S, Nieland H *et al.* Respiratory impedance in healthy subjects: Baseline values and bronchodilator response. *Eur Respir J* 2013; **42**: 1513–1523.

E12 Lum S, Stocks J, Stanojevic S, Wade A, Robinson P, Gustafsson P *et al.* Age and height dependence of lung clearance index and functional residual capacity. *Eur Respir J* 2013; **41**: 1371–1377.

E13 Piatti G, Fasano V, Cantarella G, Tarantola C. Body plethysmographic study of specific airway resistance in a sample of healthy adults. *Respirology* 2012; **17**: 976–983.

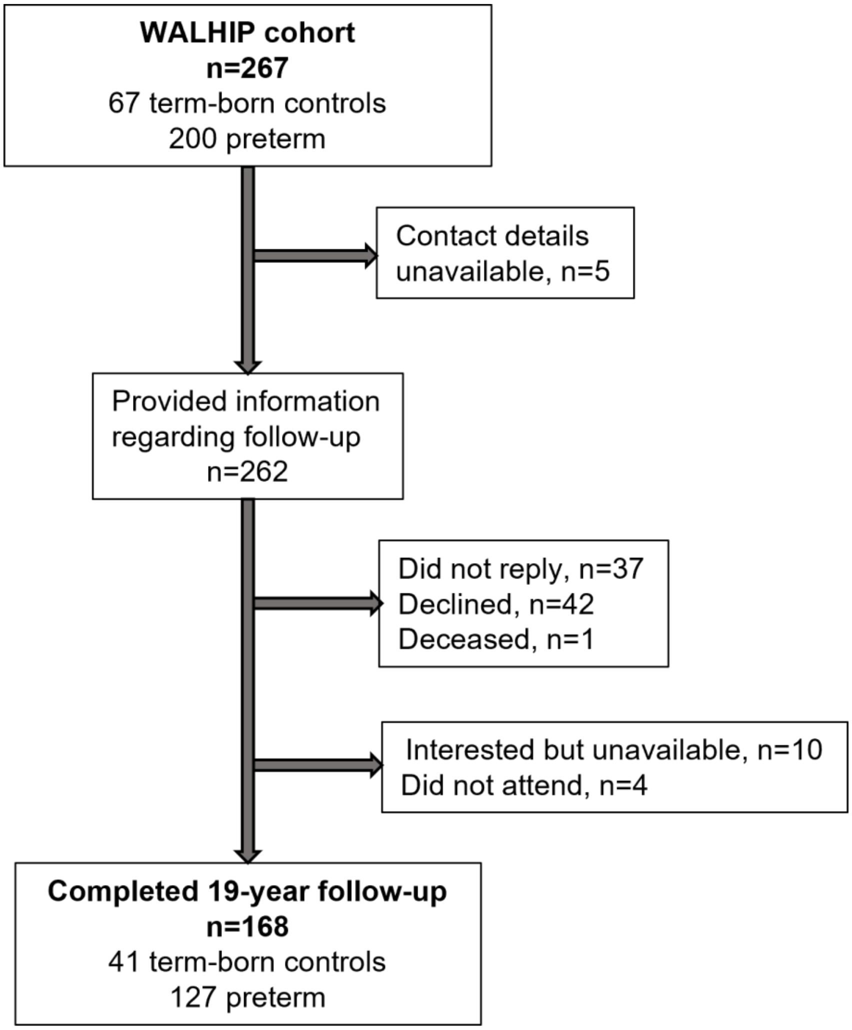

Figure E1. Flowchart describing recruitment of the WALHIP cohort at adolescent/young adulthood follow-up.

	Univariate Analysis				
	B	p-value	95% CI Lower	95% CI Upper	R^2
Forced flows and volumes					
FEV_1 z-score					
Subpleural opacities	-0.187	**<0.001***	-0.287	-0.086	0.103
Hypoattenuation – Insp	0.168	0.218	-0.437	0.101	0.013
Hypoattenuation – Exp	-0.130	**0.007***	-0.224	-0.037	0.061
Peribronchial thickening	-0.226	**0.002***	-0.369	-0.082	0.076
Total CT score	-0.102	**<0.001***	-0.144	-0.060	0.163
FEV_1 z-score post-bronchodilator					
Subpleural opacities	-0.120	**0.013***	-0.215	-0.025	0.052
Hypoattenuation – Insp	-0.102	0.402	-0.341	0.138	0.006
Hypoattenuation – Exp	0.085	0.052	-0.172	0.001	0.033
Peribronchial thickening	-0.104	0.144	-0.244	0.036	0.019
Total CT score	-0.065	**0.002***	-0.106	-0.024	0.079
FVC z-score					
Subpleural opacities	-0.041	0.409	-0.139	0.057	0.006
Hypoattenuation – Insp	-0.016	0.896	-0.263	0.230	0.000
Hypoattenuation – Exp	-0.030	0.505	-0.1520	0.059	0.004
Peribronchial thickening	0.014	0.870	-0.155	0.183	0.000
Total CT score	-0.023	0.323	-0.069	0.023	0.009
FEV_1/FVC z-score					
Subpleural opacities	-0.184	**<0.001***	-0.286	-0.082	0.104
Hypoattenuation – Insp	-0.183	0.177	-0.451	0.084	0.017
Hypoattenuation – Exp	-0.116	**0.018***	-0.211	-0.020	0.050
Peribronchial thickening	-0.203	**0.028***	-0.384	-0.022	0.043
Total CT score	-0.104	**<0.001***	-0.150	-0.058	0.152
FEF_{25-75} z-score					
Subpleural opacities	-0.229	**<0.001***	-0.331	-0.126	0.152
Hypoattenuation – Insp	-0.194	0.165	-0.469	0.081	0.017
Hypoattenuation – Exp	-0.131	**0.009***	-0.229	-0.034	0.061
Peribronchial thickening	-0.195	**0.041***	-0.382	-0.009	0.038
Total CT score	-0.120	**<0.001***	-0.167	-0.073	0.192

Table E1.The relationship between spirometry outcomes and structural abnormalities on chest CT in those born preterm. *p<0.05.

	Univariate Analysis				
	B	p-value	95% CI Lower	95% CI Upper	R^2
**DLCO z-score**					
Subpleural opacities	-0.106	**0.033***	-0.203	-0.008	0.037
Hypoattenuation – Insp	-0.288	**0.023***	-0.534	-0.041	0.043
Hypoattenuation – Exp	-0.041	0.363	-0.129	0.048	0.007
Peribronchial thickening	-0.040	0.572	-0.179	0.099	0.003
Total CT score	-0.048	**0.023***	-0.089	-0.007	0.043
**KCO z-score**					
Subpleural opacities	-0.180	**<0.001***	-0.268	-0.093	0.123
Hypoattenuation – Insp	-0.270	**0.023***	-0.502	-0.037	0.042
Hypoattenuation – Exp	0.026	0.537	-0.057	0.110	0.003
Peribronchial thickening	0.004	0.955	-0.127	0.135	0.000
Total CT score	-0.036	0.070	-0.075	0.003	0.027
**VA z-score**					
Subpleural opacities	0.087	0.108	-0.019	0.194	0.022
Hypoattenuation – Insp	-0.038	0.786	-0.311	0.236	0.001
Hypoattenuation – Exp	-0.086	0.075	-0.182	0.009	0.026
Peribronchial thickening	-0.053	0.489	-0.203	0.098	0.004
Total CT score	-0.017	0.468	-0.063	0.029	0.004

Table E2.The relationship between gas transfer (DLCO) outcomes and structural abnormalities on chest CT in those born preterm. *$p<0.05$.

	Univariate Analysis				
	B	p-value	95% CI Lower	95% CI Upper	R^2
**RV z-score**					
Subpleural opacities	0.100	**0.005***	0.032	0.169	0.066
Hypoattenuation – Insp	0.017	0.856	-0.167	0.201	0.000
Hypoattenuation – Exp	-0.068	**0.037***	-0.131	-0.004	0.036
Peribronchial thickening	0.049	0.342	-0.052	0.149	0.008
Total CT score	0.007	0.630	-0.023	0.038	0.002
**RV/TLC z-score**					
Subpleural opacities	0.095	**0.022***	0.014	0.176	0.043
Hypoattenuation – Insp	0.014	0.899	-0.201	0.228	0.000
Hypoattenuation – Exp	-0.059	0.118	-0.134	0.015	0.020
Peribronchial thickening	0.052	0.383	-0.066	0.169	0.006
Total CT score	0.010	0.594	-0.026	0.045	0.002

Table E3.The relationship between residual volume and structural abnormalities on chest CT in those born preterm. *$p<0.05$.

	Univariate Analysis				
	B	p-value	95% CI Lower	95% CI Upper	R^2
**LCI**					
Subpleural opacities	0.219	**0.004***	-0.073	0.364	0.065
Hypoattenuation – Insp	0.054	0.788	-0.345	0.453	0.001
Hypoattenuation – Exp	0.269	**<0.001***	0.158	0.381	0.153
Peribronchial thickening	0.428	**<0.001***	0.212	0.645	0.108
Total CT score	0.153	**<0.001***	0.093	0.212	0.170
**MR1**					
Subpleural opacities	0.060	**0.008***	0.016	0.103	0.055
Hypoattenuation – Insp	0.012	0.841	-0.107	0.131	0.000
Hypoattenuation – Exp	0.085	**<0.001***	0.053	0.118	0.173
Peribronchial thickening	0.114	**<0.001***	0.049	0.179	0.086
Total CT score	0.044	**<0.001***	0.027	0.062	0.161
**MR2**					
Subpleural opacities	0.523	**0.007***	0.145	0.901	0.056
Hypoattenuation – Insp	0.412	0.430	-0.617	1.441	0.005
Hypoattenuation – Exp	0.694	**<0.001***	0.406	0.982	0.152
Peribronchial thickening	1.269	**<0.001***	0.721	1.818	0.142
Total CT score	0.404	**<0.001***	0.252	0.557	0.178

Table E4.The relationship between ventilation heterogeneity and structural abnormalities on chest CT in those born preterm. *p<0.05.

	Univariate Analysis				
	B	p-value	95% CI Lower	95% CI Upper	R^2
**FeNO**					
Subpleural opacities	-0.972	0.343	-2.991	1.048	0.007
Hypoattenuation – Insp	1.007	0.704	-4.224	6.238	0.001
Hypoattenuation – Exp	-0.486	0.602	-2.328	1.356	0.002
Peribronchial thickening	4.102	**0.004***	1.313	6.891	0.065
Total CT score	0.162	0.715	-0.714	1.038	0.001

Table E5. The relationship between fractional exhaled nitric oxide (FeNO) and structural abnormalities on chest CT in those born preterm. *p<0.05.

	Univariate Analysis				
	B	p-value	95% CI Lower	95% CI Upper	R^2
**Rrs_5 z-score**					
Subpleural opacities	0.006	0.891	-0.081	0.093	0.000
Hypoattenuation – Insp	0.136	0.227	-0.086	0.359	0.012
Hypoattenuation – Exp	0.021	0.591	-0.057	0.100	0.002
Peribronchial thickening	0.029	0.639	-0.094	0.152	0.002
Total CT score	0.021	0.270	-0.013	0.058	0.010
**Fres z-score**					
Subpleural opacities	0.064	0.155	-0.025	0.154	0.017
Hypoattenuation – Insp	0.001	0.993	-0.232	0.234	0.000
Hypoattenuation – Exp	0.033	0.429	-0.049	0.115	0.005
Peribronchial thickening	0.039	0.552	-0.089	0.167	0.003
Total CT score	0.030	0.129	-0.009	0.068	0.019
**AX z-score**					
Subpleural opacities	0.039	0.346	-0.042	0.119	0.007
Hypoattenuation – Insp	0.036	0.735	-0.173	0.245	0.001
Hypoattenuation – Exp	0.032	0.392	-0.042	0.105	0.006
Peribronchial thickening	0.019	0.748	-0.097	0.134	0.001
Total CT score	0.024	0.167	-0.010	0.059	0.016
**Xrs_5 z-score**					
Subpleural opacities	0.026	0.537	-0.057	0.108	0.003
Hypoattenuation – Insp	0.075	0.486	-0.138	0.288	0.004
Hypoattenuation – Exp	0.038	0.319	-0.037	0.113	0.008
Peribronchial thickening	0.003	0.962	-0.115	0.120	0.000
Total CT score	0.025	0.173	-0.011	0.060	0.015

Table E6. The relationship between airway mechanics (oscillometry) and structural abnormalities on chest CT in those born preterm. *p<0.05.

	Univariate Analysis				
	B	p-value	95% CI Lower	95% CI Upper	R^2
sGaw					
Subpleural opacities	-0.009	**0.016***	-0.016	-0.002	0.050
Hypoattenuation – Insp	-0.010	0.267	-0.028	0.008	0.011
Hypoattenuation – Exp	-0.006	0.062	-0.013	0.000	0.030
Peribronchial thickening	-0.018	**<0.001***	-0.027	-0.008	0.097
Total CT score	-0.005	**<0.001***	-0.008	-0.002	0.092

Table E7. The relationship between airway mechanics (plethysmography) and structural abnormalities on chest CT in those born preterm. *p<0.05.

	Wheeze		Wheeze during exercise		Cough		Rattle	
	**No**	**Yes**	**No**	**Yes**	**No**	**Yes**	**No**	**Yes**
Number, N (%)	112 (88.2%)	15 (11.8%)	104 (81.9)	23 (18.1%)	63 (49.6%)	64 (50.4%)	106 (83.5%)	21 (16.5%)
R5-20	0.384 (0.613)	**1.391 (1.404)***	0.410 (0.704)	**0.924 (1.107)***	0.403 (0.680)	0.605 (0.920)	0.409 (0.672)	**0.978 (1.220)***
R5 z-score	1.234 (1.161)	**2.002 (1.103)***	1.190 (1.146)	**1.931 (1.143)***	1.178 (1.338)	1.472 (0.982)	1.239 (1.177)	1.755 (1.105)
Fres z-score	1.839 (1.431)	2.452 (1.254)	1.807 (1.401)	2.374 (1.462)	1.721 (1.522)	2.091 (1.297)	1.830 (1.439)	2.311 (1.279)
AX z-score	1.812 (1.097)	**2.731 (1.262)***	1.821 (1.103)	**2.369 (1.280)***	1.693 (1.171)	**2.148 (1.097)***	1.820 (1.099)	**2.428 (1.300)***
X5 z-score	1.135 (1.141)	**2.998 (2.482)***	1.245 (1.385)	1.864 (1.820)	1.034 (1.418)	**1.679 (1.494)***	1.168 (1.180)	**2.301 (2.324)***
FEV_1 z-score	-0.73 (1.06)	**-1.96 (1.29)***	-0.80 (1.13)	-1.21 (1.27)	-0.73 (1.27)	-1.03 (1.03)	-0.76 (1.07)	**-1.46 (1.43)***
FEV_1 z-score post-bronchodilator	-0.17 (0.98)	**-1.09 (0.99)***	-0.21 (0.99)	-0.53 (1.13)	-0.22 (1.16)	-0.33 (0.86)	-0.20 (0.99)	-0.64 (1.13)
FVC z-score	0.04 (1.02)	-0.47 (0.98)	0.01 (1.01)	0.00 (1.12)	0.07 (1.09)	-0.10 (0.95)	-0.04 (1.02)	-0.29 (1.05)
FEV_1/FVC z-score	-1.09 (1.11)	**-1.81 (1.05)***	-1.07 (1.14)	**-1.61 (0.90)***	-1.14 (1.19)	-1.20 (1.05)	-1.12 (1.14)	-1.47 (0.95)
FEF_{25-75} z-score	-1.18 (1.11)	**-2.22 (1.15)***	-1.17 (1.16)	**-1.82 (1.00)***	-1.24 (1.27)	-1.36 (1.02)	-1.22 (1.15)	-1.74 (1.13)
FEF_{75} z-score	-0.81 (1.03)	**-1.69 (1.15)***	-0.81 (1.07)	**-1.34 (0.99)***	-0.88 (1.15)	-0.94 (0.98)	-0.82 (1.05)	**-1.39 (1.09)***
DLCO z-score	0.87 (1.11)	0.76 (0.83)	0.90 (1.09)	0.64 (1.03)	1.01 (1.14)	0.70 (1.01)	0.93 (1.09)	0.44 (0.97)
VA z-score	0.71 (1.17)	0.11 (1.19)	0.70 (1.17)	0.37 (1.24)	0.82 (1.21)	0.47 (1.13)	0.79 (1.18)	**-0.14 (0.87)***
KCO z-score	0.29 (0.98)	0.68 (1.20)	0.34 (0.98)	0.33 (1.16)	0.35 (1.02)	0.32 (1.02)	0.29 (0.99)	0.55 (1.11)
TLC z-score	0.30 (0.82)	0.17 (0.87)	0.30 (0.82)	0.19 (0.84)	0.40 (0.85)	0.17 (0.78)	0.35 (0.82)	**-0.06 (0.74)***
FRC z-score	0.53 (0.89)	0.71 (1.10)	0.55 (0.92)	0.54 (0.92)	0.57 (0.86)	0.52 (0.98)	0.57 (0.85)	0.43 (1.21)
RV z-score	0.65 (0.77)	0.93 (0.96)	0.68 (0.81)	0.70 (0.72)	0.68 (0.83)	0.70 (0.76)	0.69 (0.75)	0.67 (1.01)
RV/TLC z-score	0.72 (0.90)	1.13 (1.07)	0.76 (0.94)	0.82 (0.88)	0.71 (0.96)	0.83 (0.90)	0.75 (0.89)	0.88 (1.11)
**Median (IQR)**								
FeNO (ppb)	15 (10,24)	17 (14, 45)	15 (10, 26)	16 (10, 23)	15 (11,27)	16 (10, 23)	15 (10, 26)	16 (13, 19)
sGAW	0.16 (0.12, 0.23)	**0.11 (0.05, 0.14)**	0.16 (0.12, 0.23)	**0.12 (0.10, 0.15)**	0.16 (0.11, 0.24)	0.15 (0.11, 0.20)	0.15 (0.12, 0.23)	0.14 (0.08, 0.20)
LCI	7.45 (6.88, 8.22)	7.88 (7.27, 9.99)	7.60 (6.88, 8.22)	7.48 (7.02, 9.32)	7.44 (6.78, 8.42)	7.64 (6.96, 8.22)	7.44 6.88, 8.03)	**8.20 (7.20, 10.15)**
Moment Ratio 1	1.96 (1.67, 2.15)	2.01 (1.72, 2.38)	1.99 (1.72, 2.19)	1.93 (1.64, 2.18)	2.06 (1.68, 2.17)	1.94 (1.63, 2.28)	1.94 (1.65, 2.15)	2.26 (1.80, 2.57)
Moment Ratio 2	7.03 (5.45, 8.85)	7.58 (6.17, 11.00)	7.18 (5.61, 9.57)	6.92 (5.26, 10.27)	7.56 (5.37, 9.84)	6.96 (5.61, 9.80)	6.98 (5.36, 8.74)	9.69 (6.66, 11.72)

Table E8. The relationship between lung function and respiratory symptoms. Data expressed as mean (SD), unless otherwise indicated. *p<0.05 compared to no [symptom] group.

	Wheeze		Wheeze during exercise		Cough		Rattle	
	**No (n=110)**	**Yes (n=15)**	**No (n=102)**	**Yes (n=23)**	**No (n=62)**	**Yes (n=63)**	**No (n=105)**	**Yes (n=20)**
Linear/triangular subpleural opacities								
Presence, n participants (%)	74 (67.3%)	10 (66.7%)	67 (65.7%)	17 (73.9%)	40 (64.5%)	44 (69.8%)	71 (67.6%)	13 (65.0%)
Extent (CT score) (IQR)	2 (0,4)	2 (0,3)	2 (0,4)	2 (0,3)	2 (0,3)	2 (0,4)	2 (0,4)	2 (0,3)
Decreased pulmonary attenuation—inspiration								
Presence	12 (10.9%)	2 (13.3%)	12 (11.8%)	2 (8.7%)	5 (8.1%)	9 (14.3%)	11 (10.5%)	3 (15.0%)
Extent	0 (0,0)	0 (0,0)	0 (0,0)	0 (0,0)	0 (0,0)	0 (0,0)	0 (0,0)	0 (0,0)
Decreased pulmonary attenuation—expiration								
Presence	74 (67.3%)	12 (80.0%)	68 (66.7%)	18 (78.3%)	41 (66.1%)	45 (71.4%)	69 (65.7%)	17 (85.0%)
Extent	2 (0,4)	2 (1,5)	2 (0,4)	2 (1,4)	2 (0,4)	2 (0,5)	2 (0,4)	4 (1,6)
Decreased bronchial: arterial ratio								
Presence	3 (2.7%)	0 (0%)	3 (2.9%)	0 (0%)	3 (4.8%)	0 (0%)	3 (2.9%)	0 (0%)
Extent	0 (0,0)	0 (0,0)	0 (0,0)	0 (0,0)	0 (0,0)	0 (0,0)	0 (0,0)	0 (0,0)
Bronchiectasis								
Presence	4 (3.6%)	1 (6.7%)	5 (4.9%)	0 (0%)	3 (4.8%)	2 (3.2%)	4 (3.8%)	1 (5.0%)
Extent	0 (0,0)	0 (0,0)	0 (0,0)	0 (0,0)	0 (0,0)	0 (0,0)	0 (0,0)	0 (0,0)
Bronchial wall thickening								
Presence	11 (10.0%)	**7 (46.7%)***	12 (11.8%)	6 (26.1%)	6 (9.7%)	12 (19.0%)	11 (10.5%)	**7 (35.0%)***
Extent	0 (0,0)	**0 (0,5)***	0 (0,0)	0 (0,0)	0 (0,0)	0 (0,0)	0 (0,0)	**0 (0,3)***
Bullae								
Presence	0 (0%)	0 (0%)	0 (0%)	0 (0%)	0 (0%)	0 (0%)	0 (0%)	0 (0%)
Extent	0 (0,0)	0 (0,0)	0 (0,0)	0 (0,0)	0 (0,0)	0 (0,0)	0 (0,0)	0 (0,0)
Emphysema								
Presence	3 (2.7%)	1 (6.7%)	4 (3.9%)	0 (0%)	1 (1.6%)	3 (4.8%)	3 (2.9%)	1 (5.0%)
Extent	0 (0,0)	0 (0,0)	0 (0,0)	0 (0,0)	0 (0,0)	0 (0,0)	0 (0,0)	0 (0,0)
Collapse/consolidation								
Presence	7 (6.4%)	2 (13.3%)	7 (6.9%)	2 (8.7%)	1 (1.6%)	**8 (12.7%)***	5 (4.8%)	**4 (20.0%)***
Extent	0 (0,0)	0 (0,0)	0 (0,0)	0 (0,0)	0 (0,0)	**0 (0,0)***	0 (0,0)	**0 (0,0)***
**Structural abnormalities on chest CT**								
**Presence, n participants (%)**	95 (86.4%)	15 (100%)	87 (85.3%)	23 (100%)	52 (83.9%)	54 (92.1%)	91 (86.7%)	19 (95.0%)
**Total CT score**	4 (1,8)	5 (2,10)	4 (1,8)	5 (2,8)	4 (1,8)	5 (2,8)	4 (1,8)	6 (3,11)

Table E9. The relationship between lung structure abnormalities and respiratory symptoms. Data expressed as n (%) and median (IQR). *p<0.05 compared to no [symptom] group.

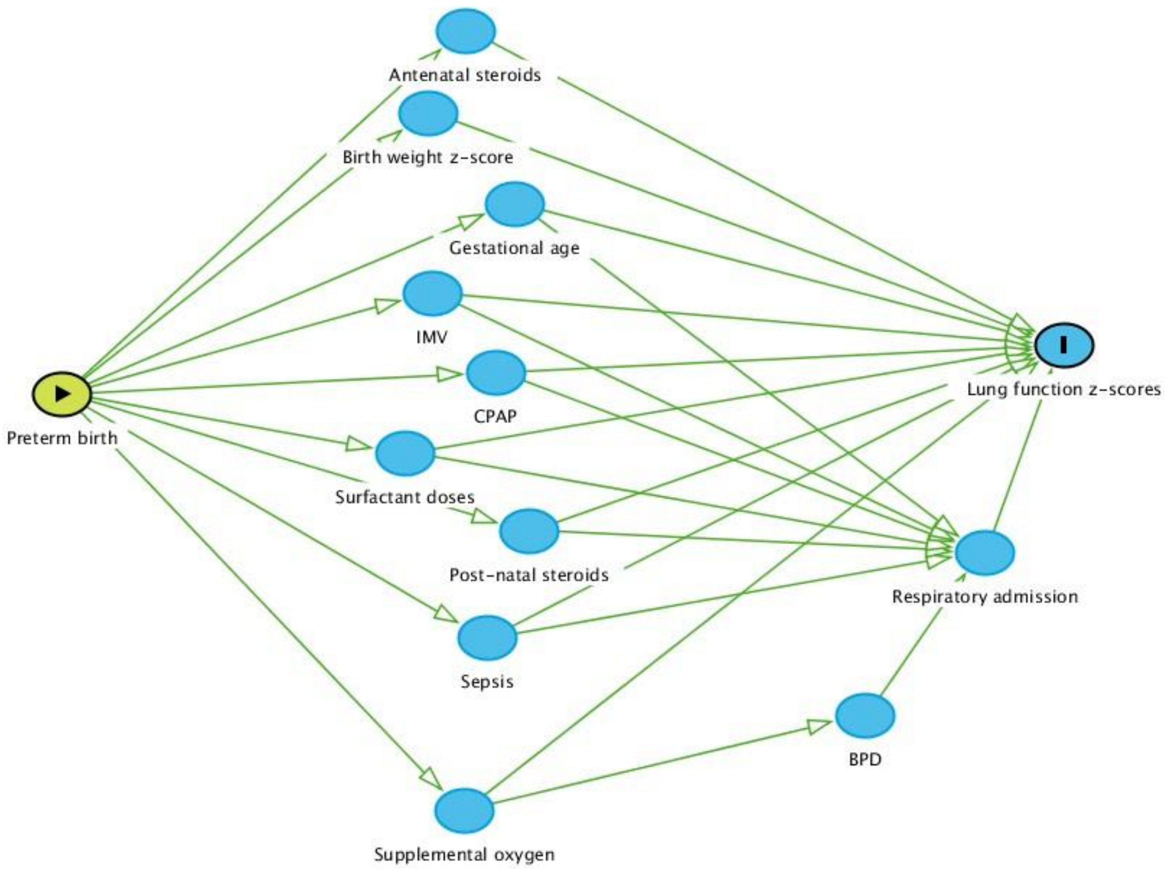

Figure E2. Direct acyclic graph for relationships between Post-natal/early life factors and lung function in children born preterm. Lung function outcomes are the outcome variable in blue and preterm birth the exposure variable (green)). Arrows in green denote causal paths.

	Univariate Analysis				Multivariable Linear Regression Analysis				
	B	p-value	95% CI Lower	95% CI Upper	B	p-value	95% CI Lower	95% CI Upper	Model R^2 change
**FEV_1 z-score**									$R^{2=}$0.101; p=0.148
Antenatal Steroids (y/n)	-0.006	0.316	-0.18	0.006					
Birth weight z-score	0.265	**0.029***	0.028	0.503					
Gestation Age (w)	0.098	**0.024***	0.013	0.183	-0.022	0.797	-0.189	0.145	0.048
IMV (d)	-0.014	**0.006***	-0.025	-0.004	-0.005	0.655	-0.026	0.017	0.014
Supplementary O2 (d)	-0.007	**0.002***	-0.011	-0.003	-0.005	0.296	-0.014	0.004	0.016
CPAP (d)	-0.009	0.267	-0.024	0.007	-0.001	0.919	-0.022	0.020	0.000
Surfactant doses (n)	-0.087	0.476	-0.327	0.154	0.069	0.608	-0.197	0.335	0.002
Post-natal steroids (y/n)	-0.418	0.078	-0.884	0.048	-0.034	0.910	-0.627	0.559	0.000
Sepsis (n)	-0.364	0.108	-0.810	0.081	-0.182	0.470	-0.681	0.316	0.003
Respiratory admission (y/n)	-0.435	**0.039***	-0.848	-0.022	-0.320	0.142	-0.749	0.109	0.018
**FEV_1 z-score post-bronchodilator**									$R^{2=}$0.062; p=0.222
Antenatal Steroids (y/n)	-0.007	0.200	-0.017	-0.004					
Birth weight z-score	0.262	0.018	0.045	0.479					
Gestation Age (w)	0.064	0.104	-0.013	0.141	0.000	0.998	-0.152	0.152	0.027
IMV (d)	-0.008	0.128	-0.017	0.002	0.005	0.611	-0.015	0.025	0.000
Supplementary O2 (d)	-0.005	**0.014***	-0.008	-0.001	-0.006	0.136	-0.014	0.002	0.024
CPAP (d)	-0.009	0.222	-0.022	0.005	0.000	0.990	-0.020	0.020	0.000
Surfactant doses (n)	-0.004	0.974	-0.217	0.210	0.113	0.355	-0.128	0.354	0.007
Post-natal steroids (y/n)	-0.223	0.304	-0.651	0.205	-0.032	0.908	-0.577	0.513	0.000
Sepsis (n)	-0.247	0.223	-0.646	0.152	-0.127	0.579	-0.579	0.325	0.002
Respiratory admission (y/n)	-0.166	0.380	-0.539	0.207	-0.071	0.719	-0.464	0.321	0.001
**FVC z-score**									R^2=0.072; p=0.444
Antenatal Steroids (y/n)	-0.006	0.319	-0.018	0.006					
Birth weight z-score	0.221	0.050	0.000	0.441					
Gestation Age (w)	0.048	0.223	-0.030	0.126	-0.036	0.645	-0.192	0.119	0.017
IMV (d)	-0.008	0.130	-0.017	0.002	-0.006	0.575	-0.028	0.016	0.003
Supplementary O2 (d)	-0.004	0.062	-0.007	0.000	-0.003	0.568	-0.011	0.006	0.008
CPAP (d)	-0.011	0.100	-0.025	0.002	-0.014	0.183	-0.035	0.007	0.015
Surfactant doses (n)	0.060	0.586	-0.157	0.276	0.193	0.121	-0.052	0.437	0.020
Post-natal steroids (y/n)	-0.235	0.289	-0.671	0.202	-0.104	0.714	-0.662	0.455	0.002
Sepsis (n)	-0.161	0.442	-0.576	0.253	-0.007	0.975	-0.473	0.458	0.000
Respiratory admission (y/n)	0.100	0.606	-0.283	0.483	0.183	0.372	-0.222	0.589	0.007
**FEV_1/FVC z-score**									R^2=0.113; p=0.126
Antenatal Steroids (y/n)	-0.009	0.187	-0.022	0.004					
Birth weight z-score	0.115	0.355	-0.130	0.359					
Gestation Age (w)	0.051	0.242	-0.035	0.136	0.018	0.833	-0.149	0.185	0.015
IMV (d)	-0.006	0.277	-0.017	0.005	0.012	0.295	-0.011	0.036	0.000
Supplementary O2 (d)	-0.004	0.079	-0.008	0.000	-0.005	0.283	-0.015	0.004	0.013
CPAP (d)	0.000	0.951	-0.015	0.016	0.014	0.220	-0.008	0.036	0.018
Surfactant doses (n)	-0.143	0.232	-0.378	0.093	-0.106	0.426	-0.369	0.157	0.006
Post-natal steroids (y/n)	-0.264	0.276	-0.741	0.213	-0.169	0.577	-0.769	0.431	0.001
Sepsis (n)	-0.214	0.354	-0.670	0.242	-0.265	0.295	-0.766	0.235	0.004
Respiratory admission (y/n)	-0.613	**0.003***	-1.016	-0.210	-0.561	**0.012***	-0.998	-0.125	0.057

**FEF_{25-75} z-score**									R^2=0.125; p=0.082
Antenatal Steroids (y/n)	-0.011	0.095	-0.025	0.002					
Birth weight z-score	0.277	**0.029***	0.029	0.525					
Gestation Age (w)	0.076	0.088	-0.012	0.164	-0.016	0.852	-0.186	0.154	0.032
IMV (d)	-0.011	0.058	-0.022	0.000	0.007	0.555	-0.017	0.031	0.004
Supplementary O2 (d)	-0.006	**0.007***	-0.010	-0.002	-0.007	0.146	-0.017	0.003	0.027
CPAP (d)	-0.004	0.585	-0.020	0.011	0.009	0.447	-0.014	0.032	0.007
Surfactant doses (n)	-0.127	0.302	-0.371	0.116	-0.030	0.823	-0.298	0.237	0.001
Post-natal steroids (y/n)	-0.320	0.200	-0.811	0.171	-0.088	0.775	-0.699	0.523	0.000
Sepsis (n)	-0.316	0.181	-0.781	0.149	-0.289	0.263	-0.798	0.220	0.005
Respiratory admission (y/n)	-0.629	**0.003***	-1.045	-0.213	-0.535	**0.019***	-0.979	-0.090	0.049

Table E10. Neonatal factors influencing forced flows and volumes in preterm adolescents and young adults. *p<0.05.

	Univariate Analysis				Multivariable Linear Regression Analysis				
	B	p-value	95% CI Lower	95% CI Upper	B	p-value	95% CI Lower	95% CI Upper	Model R^2 change
**DLCO z-score**									R^2=0.054; p=0.604
Antenatal Steroids (y/n)	-0.012	0.065	-0.025	0.001					
Birth weight z-score	0.172	0.115	-0.042	0.386					
Gestation Age (w)	0.045	0.262	-0.034	0.124	-0.032	0.689	-0.193	0.128	0.012
IMV (d)	-0.008	0.129	-0.017	0.002	-0.003	0.793	-0.023	0.017	0.006
Supplementary O2 (d)	-0.004	0.068	-0.008	0.000	-0.005	0.225	-0.013	0.003	0.010
CPAP (d)	-0.004	0.618	-0.018	0.011	-0.003	0.779	-0.023	0.018	0.000
Surfactant doses (n)	-0.056	0.617	-0.275	0.164	0.006	0.962	-0.247	0.259	0.000
Post-natal steroids (y/n)	-0.274	0.219	-0.714	0.165	-0.063	0.829	-0.638	0.512	0.001
Sepsis (n)	0.196	0.356	-0.223	0.616	0.411	0.090	-0.066	0.887	0.024
Respiratory admission (y/n)	-0.037	0.849	-0.425	0.351	0.079	0.704	-0.332	0.489	0.001
**KCO z-score**									R^2=0.117; p=0.073
Antenatal Steroids (y/n)	-0.004	0.464	-0.016	0.534					
Birth weight z-score	0.079	0.442	-0.123	0.280					
Gestation Age (w)	0.033	0.382	-0.041	0.106	-0.098	0.182	-0.243	0.047	0.006
IMV (d)	-0.009	0.052	-0.018	0.000	-0.005	0.549	-0.024	0.013	0.035
Supplementary O2 (d)	-0.003	0.066	-0.007	0.000	-0.005	0.177	-0.013	0.002	0.015
CPAP (d)	0.003	0.700	-0.011	0.016	0.005	0.590	-0.014	0.024	0.002
Surfactant doses (n)	-0.287	**0.005***	-0.485	-0.088	-0.295	**0.012***	-0.523	-0.066	0.047
Post-natal steroids (y/n)	-0.300	0.150	-0.710	0.110	-0.009	0.973	-0.528	0.510	0.000
Sepsis (n)	0.072	0.717	-0.321	0.466	0.274	0.209	-0.156	0.704	0.013
Respiratory admission (y/n)	-0.061	0.740	-0.423	0.302	-0.022	0.905	-0.393	0.348	0.000
**VA z-score**									R^2=0.089; p=0.222
Antenatal Steroids (y/n)	-0.010	0.148	-0.024	0.004					
Birth weight z-score	0.115	0.336	-0.120	0.349					
Gestation Age (w)	0.017	0.700	-0.069	0.103	0.081	0.348	-0.089	0.251	0.002
IMV (d)	0.002	0.700	-0.009	0.013	0.004	0.686	-0.017	0.026	0.015
Supplementary O2 (d)	0.000	0.854	-0.005	0.004	0.000	0.945	-0.009	0.008	0.000
CPAP (d)	-0.009	0.255	-0.024	0.006	-0.011	0.334	-0.032	0.011	0.002
Surfactant doses (n)	0.282	**0.018***	0.048	0.516	0.371	**0.007***	0.103	0.639	0.063
Post-natal steroids (y/n)	0.049	0.841	-0.433	0.531	-0.053	0.864	-0.662	0.557	0.000
Sepsis (n)	0.138	0.549	-0.317	0.592	0.161	0.528	-0.344	0.666	0.003
Respiratory admission (y/n)	0.025	0.907	-0.398	0.448	0.126	0.569	-0.310	0.561	0.003

Table E11. Neonatal factors influencing gas transfer in preterm adolescents and young adults. *$p<0.05$.

	Univariate Analysis				Multivariable Linear Regression Analysis				
	B	p-value	95% CI Lower	95% CI Upper	B	p-value	95% CI Lower	95% CI Upper	Model R^2 change
**RV z-score**									**R^2=0.133; p=0.037**
Antenatal Steroids (y/n)	-0.001	0.842	-0.009	0.007					
Birth weight z-score	-0.174	**0.029***	-0.330	-0.019					
Gestation Age (w)	-0.051	0.075	-0.108	0.005	0.105	0.067	-0.007	0.217	0.028
IMV (d)	0.012	**<0.001***	0.005	0.019	0.016	**0.026***	0.002	0.030	0.066
Supplementary O2 (d)	0.004	**0.003***	0.001	0.007	0.002	0.506	-0.004	0.008	0.011
CPAP (d)	0.006	0.244	-0.004	0.016	0.008	0.263	-0.006	0.023	0.016
Surfactant doses (n)	0.190	**0.019***	0.031	0.348	0.105	0.249	-0.075	0.285	0.010
Post-natal steroids (y/n)	0.293	0.066	-0.020	0.607	-0.076	0.706	-0.477	0.324	0.001
Sepsis (n)	0.223	0.149	-0.081	0.528	0.007	0.968	-0.326	0.340	0.000
Respiratory admission (y/n)	-0.010	0.946	-0.294	0.275	-0.051	0.727	-0.340	0.238	0.001
**RV/TLC z-score**									**R^2=0.146; p=0.020**
Antenatal Steroids (y/n)	0.001	0.904	-0.009	0.010					
Birth weight z-score	-0.281	**0.002***	-0.460	-0.103					
Gestation Age (w)	-0.065	0.054	-0.131	0.001	0.132	**0.045***	0.003	0.262	0.035
IMV (d)	0.014	**<0.001***	0.006	0.022	0.019	**0.024***	0.002	0.035	0.059
Supplementary O2 (d)	0.005	**0.001***	0.002	0.009	0.003	0.401	-0.004	0.010	0.018
CPAP (d)	0.011	0.076	-0.001	0.023	0.015	0.081	-0.002	0.031	0.030
Surfactant doses (n)	0.180	0.059	-0.007	0.367	0.048	0.647	-0.159	0.255	0.002
Post-natal steroids (y/n)	0.385	**0.039***	0.021	0.751	-0.024	0.918	-0.486	0.438	0.000
Sepsis (n)	0.290	0.106	-0.063	0.643	0.014	0.944	-0.370	0.398	0.000
Respiratory admission (y/n)	-0.022	0.895	-0.355	0.310	-0.089	0.598	-0.423	0.245	0.002

Table E12. Neonatal factors influencing residual volume in preterm adolescents and young adults. *p<0.05.

	Univariate Analysis				Multivariable Linear Regression Analysis				
	B	p-value	95% CI Lower	95% CI Upper	B	p-value	95% CI Lower	95% CI Upper	Model R^2 change
**Rrs5 z-score**									R^2=0.091; p=0.192
Antenatal Steroids (y/n)	0.007	0.272	-0.005	0.019					
Birth weight z-score	0.093	0.431	-0.141	0.327					
Gestation Age (w)	-0.080	0.063	-0.164	0.004	-0.166	0.054	-0.335	0.003	0.029
IMV (d)	0.002	0.691	-0.008	0.013	-0.009	0.405	-0.030	0.012	0.014
Supplementary O2 (d)	0.003	0.171	-0.001	0.007	0.004	0.339	-0.005	0.013	0.001
CPAP (d)	0.006	0.425	-0.009	0.021	-0.010	0.354	-0.032	0.011	0.008
Surfactant doses (n)	-0.066	0.582	-0.303	0.171	-0.148	0.279	-0.418	0.121	0.014
Post-natal steroids (y/n)	-0.258	0.276	-0.726	0.209	-0.520	0.092	-1.127	0.087	0.022
Sepsis (n)	-0.036	0.876	-0.490	0.418	-0.149	0.560	-0.653	0.355	0.003
Respiratory admission (y/n)	0.134	0.526	-0.283	0.552	-0.037	0.868	-0.471	0.398	0.000
**Fres z-score**									**R^2=0.133; p=0.037**
Antenatal Steroids (y/n)	0.007	0.337	-0.007	0.022					
Birth weight z-score	-0.197	0.169	-0.479	0.085					
Gestation Age (w)	-0.156	**0.002***	-0.256	-0.057	-0.178	0.079	-0.376	0.021	0.078
IMV (d)	0.012	0.056	0.000	0.024	-0.013	0.303	-0.038	0.012	0.003
Supplementary O2 (d)	0.008	**0.002***	0.003	0.013	0.008	0.127	-0.002	0.018	0.013
CPAP (d)	0.012	0.183	-0.006	0.031	-0.014	0.267	-0.040	0.011	0.010
Surfactant doses (n)	-0.002	0.989	-0.292	0.288	-0.223	0.171	-0.544	0.098	0.016
Post-natal steroids (y/n)	0.159	0.582	-0.410	0.728	-0.288	0.427	-1.004	0.428	0.006
Sepsis (n)	0.429	0.121	-0.114	0.973	0.235	0.436	-0.359	0.828	0.004
Respiratory admission (y/n)	0.403	0.116	-0.101	0.908	0.182	0.488	-0.336	0.699	0.004
**AX z-score**									R^2=0.117; p=0.068
Antenatal Steroids (y/n)	0.003	0.635	-0.009	0.015					
Birth weight z-score	-0.135	0.245	-0.363	0.094					
Gestation Age (w)	-0.106	0.011	-0.187	-0.025	-0.126	0.128	-0.288	0.037	0.054
IMV (d)	0.008	0.125	-0.002	0.018	-0.009	0.396	-0.029	0.012	0.003
Supplementary O2 (d)	0.005	**0.009***	0.001	0.009	0.006	0.148	-0.002	0.015	0.010
CPAP (d)	0.007	0.336	-0.008	0.022	-0.012	0.256	-0.033	0.009	0.012
Surfactant doses (n)	-0.031	0.792	-0.264	0.201	-0.175	0.185	-0.435	0.085	0.017
Post-natal steroids (y/n)	0.027	0.908	-0.433	0.487	-0.321	0.280	-0.906	0.264	0.010
Sepsis (n)	0.228	0.308	-0.214	0.670	0.114	0.644	-0.372	0.600	0.001
Respiratory admission (y/n)	0.401	0.051	-0.002	0.804	0.247	0.246	-.0172	0.666	0.011
**Xrs5 z-score**									R^2=0.107; p=0.105
Antenatal Steroids (y/n)	-0.002	0.817	-0.017	0.013					
Birth weight z-score	-0.120	0.424	-0.415	0.176					
Gestation Age (w)	-0.121	**0.025***	-0.226	-0.016	-0.076	0.479	-0.287	0.136	0.042
IMV (d)	0.011	0.090	-0.002	0.024	-0.004	0.746	-0.031	0.022	0.000
Supplementary O2 (d)	0.007	**0.007***	0.002	0.012	0.010	0.084	-0.001	0.021	0.018
CPAP (d)	0.008	0.393	-0.011	0.028	-0.013	0.337	-0.040	0.014	0.008
Surfactant doses (n)	0.011	0.940	-0.289	0.312	-0.151	0.378	-0.488	0.187	0.009
Post-natal steroids (y/n)	-0.028	0.926	-0.622	0.566	-0.583	0.131	-1.343	0.177	0.019
Sepsis (n)	0.268	0.354	-0.303	0.839	0.083	0.796	-0.549	0.714	0.000
Respiratory admission (y/n)	0.505	0.057	-0.016	1.025	0.317	0.252	-0.228	0.861	0.010

Table E13. Neonatal factors influencing airway mechanics (oscillometry) in preterm adolescents and young adults. *p<0.05.

	Univariate Analysis				Multivariable Linear Regression Analysis				
	B	p-value	95% CI Lower	95% CI Upper	B	p-value	95% CI Lower	95% CI Upper	Model R^2 change
**Specific Airway Conductance (sGaw)**									R^2=0.076; p=0.367
Antenatal Steroids (y/n)	0.000	0.631	-0.001	0.001					
Birth weight z-score	0.005	0.538	-0.011	0.021					
Gestation Age (w)	0.004	0.149	-0.002	0.010	0.002	0.747	-0.010	0.013	0.021
IMV (d)	-0.001	0.084	-0.001	0.000	0.000	0.916	-0.001	0.002	0.007
Supplementary O2 (d)	0.000	0.058	-0.001	0.000	0.000	0.345	-0.001	0.000	0.004
CPAP (d)	0.000	0.615	-0.001	0.001	0.001	0.212	-0.001	0.002	0.019
Surfactant doses (n)	-0.007	0.374	-0.023	0.009	-0.004	0.675	-0.023	0.015	0.001
Post-natal steroids (y/n)	-0.017	0.290	-0.049	0.015	0.005	0.817	-0.037	0.047	0.001
Sepsis (n)	0.000	0.982	-0.031	0.030	0.004	0.805	-0.030	0.039	0.002
Respiratory admission (y/n)	-0.032	**0.024**	-0.060	-0.004	-0.024	0.111	-0.054	0.006	0.022

Table E14. Neonatal factors influencing airway mechanics (plethysmography) in preterm adolescents and young adults. *p<0.05.

	Univariate Analysis				Multivariable Linear Regression Analysis				
	B	p-value	95% CI Lower	95% CI Upper	B	p-value	95% CI Lower	95% CI Upper	Model R^2 change
**Fractional Exhaled Nitric Oxide (FeNO)**									**R^2=0.140; p=0.024**
Antenatal Steroids (y/n)	-0.070	0.547	-0.300	0.160					
Birth weight z-score	2.663	0.237	-1.775	7.101					
Gestation Age (w)	2.280	**0.005***	0.712	3.849	1.353	0.393	-1.771	4.476	0.066
IMV (d)	-0.270	**0.006***	-0.462	-0.077	0.097	0.624	-0.294	0.489	0.007
Supplementary O2 (d)	-0.126	**0.001**	-0.202	-0.049	-0.111	0.178	-0.273	0.051	0.009
CPAP (d)	-0.123	0.403	-0.413	0.167	0.251	0.217	-0.150	0.652	0.005
Surfactant doses (n)	-6.647	**0.003***	-11.009	-2.284	-4.344	0.087	-9.335	0.646	0.027
Post-natal steroids (y/n)	-8.470	0.060	-17.288	0.347	0.317	0.956	-10.921	11.555	0.000
Sepsis (n)	-7.319	0.092	-15.852	1.214	-1.280	0.786	-10.611	8.052	0.002
Respiratory admission (y/n)	4.171	0.300	-3.756	12.098	7.383	0.072	-0.666	15.431	0.025

Table E15. Neonatal factors influencing airway inflammation (fractional exhaled nitric oxide (FeNO) in preterm adolescents and young adults. *p<0.05.

	Univariate Analysis				Multivariable Linear Regression Analysis				
	B	p-value	95% CI Lower	95% CI Upper	B	p-value	95% CI Lower	95% CI Upper	Model R^2 change
**Lung Clearance Index (LCI)**									R^2=0.065; p=0.647
Antenatal Steroids (y/n)	-0.004	0.806	-0.037	0.029					
Birth weight z-score	-0.314	0.106	-0.696	0.068					
Gestation Age (w)	-0.083	0.215	-0.216	0.049	-0.074	0.572	-0.334	0.186	0.016
IMV (d)	0.013	0.173	-0.00	0.032	0.000	0.996	-0.042	0.042	0.006
Supplementary O2 (d)	0.004	0.311	-0.003	0.011	-0.001	0.904	-0.018	0.016	0.002
CPAP (d)	0.001	0.946	-0.022	0.024	-0.013	0.504	-0.050	0.025	0.002
Surfactant doses (n)	0.116	0.514	-0.236	0.468	0.012	0.952	-0.391	0.416	0.000
Post-natal steroids (y/n)	0.232	0.575	-0.588	1.053	0.030	0.953	-0.977	1.038	0.000
Sepsis (n)	0.785	**0.031***	0.075	1.495	0.802	0.063	-0.061	1.665	0.035
Respiratory admission (y/n)	0.312	0.259	-0.232	0.855	0.218	0.529	-0.468	0.905	0.004
**Moment Ratio 1 (MR1)**									R^2=0.069; p=0.597
Antenatal Steroids (y/n)	-0.004	0.426	-0.014	0.006					
Birth weight z-score	-0.049	0.392	-0.164	0.065					
Gestation Age (w)	-0.025	0.205	-0.065	0.014	-0.037	0.340	-0.114	0.040	0.017
IMV (d)	0.005	0.107	-0.001	0.010	0.001	0.831	-0.011	0.014	0.011
Supplementary O2 (d)	0.001	0.444	-0.001	0.003	-0.001	0.552	-0.006	0.003	0.013
CPAP (d)	0.001	0.757	-0.008	0.006	-0.004	0.473	-0.015	0.007	0.002
Surfactant doses (n)	0.032	0.549	-0.073	0.136	0.003	0.961	-0.122	0.116	0.000
Post-natal steroids (y/n)	0.155	0.206	-0.087	0.396	0.085	0.574	-0.213	0.383	0.002
Sepsis (n)	0.203	0.059	-0.008	0.415	0.192	0.139	-0.064	0.447	0.024
Respiratory admission (y/n)	0.041	0.619	-0.121	0.202	0.004	0.968	-0.199	0.207	0.000
**Moment Ratio 2 (MR2)**									R^2=0.060; p=0.699
Antenatal Steroids (y/n)	-0.027	0.534	-0.113	0.059					
Birth weight z-score	-0.564	0.268	-1.570	0.442					
Gestation Age (w)	-0.203	0.248	-0.550	0.144	-0.262	0.448	-0.943	0.420	0.014
IMV (d)	0.037	0.136	-0.012	0.086	0.014	0.798	-0.096	0.124	0.010
Supplementary O2 (d)	0.007	0.459	-0.012	0.026	-0.011	0.605	-0.055	0.032	0.009
CPAP (d)	-0.010	0.738	-0.070	0.050	-0.038	0.447	-0.135	0.060	0.003
Surfactant doses (n)	0.474	0.307	-0.443	1.391	0.299	0.575	-0.758	1.357	0.005
Post-natal steroids (y/n)	1.031	0.341	-1.107	3.169	0.325	0.808	-2.318	2.967	0.000
Sepsis (n)	1.540	0.107	-0.337	3.416	1.412	0.218	-0.851	3.676	0.015
Respiratory admission (y/n)	0.758	0.284	-0.637	2.153	0.551	0.544	-1.248	2.349	0.004

Table E16. Neonatal factors influencing ventilation heterogeneity in preterm adolescents and young adults. *$p<0.05$.

	Negative Binomial Univariate Regression Analysis				Negative Binomial Multi-variable Regression Analysis			
	IRR	p-value	95% CI Lower	95% CI Upper	IRR	p-value	95% CI Lower	95% CI Upper
**Subpleural opacities**								
Antenatal Steroids (y/n)	1.066	0.824	0.606	1.877				
Birth weight z-score	1.134	0.316	0.887	1.450				
Gestation Age (w)	0.855	**0.001***	0.777	0.941	0.955	0.634	0.792	1.153
IMV (d)	1.014	**0.012***	1.003	1.025	0.998	0.817	0.977	1.019
Supplementary O2 (d)	1.008	**<0.001***	1.003	1.012	1.005	0.267	0.996	1.014
CPAP (d)	1.014	0.092	0.998	1.031	0.998	0.843	0.975	1.020
Surfactant doses (n)	1.416	**0.008***	1.096	1.829	1.242	0.135	0.935	1.649
Post-natal steroids (y/n)	1.379	0.182	0.861	2.208	0.857	0.630	0.457	1.606
Sepsis (n)	1.799	**0.011***	1.142	2.834	1.346	0.257	0.805	2.249
Respiratory admission (y/n)	1.326	0.199	0.862	2.040	1.190	0.459	0.751	1.886
**Hypoattenuation – Inspiration**								
Antenatal Steroids (y/n)	1.000	1.000	0.000	0.000				
Birth weight z-score	1.933	**0.020***	1.110	3.366				
Gestation Age (w)	0.909	0.286	0.764	1.083	0.951	0.834	0.594	1.522
IMV (d)	1.004	0.655	0.985	1.024	0.962	0.105	0.917	1.008
Supplementary O2 (d)	1.007	0.109	0.999	1.015	1.018	0.066	0.999	1.037
CPAP (d)	1.001	0.961	0.973	1.029	0.975	0.256	0.934	1.018
Surfactant doses (n)	3.272	**0.004***	1.457	7.350	2.931	**0.006***	1.352	6.356
Post-natal steroids (y/n)	1.309	0.554	0.536	3.195	0.923	0.906	0.245	3.486
Sepsis (n)	0.280	**0.047***	0.079	0.985	0.191	**0.023***	0.046	0.796
Respiratory admission (y/n)	3.675	**0.009***	1.388	9.731	2.511	0.091	0.864	7.300
**Hypoattenuation – Expiration**								
Antenatal Steroids (y/n)	1.571	0.128	0.878	2.814				
Birth weight z-score	1.023	0.855	0.805	1.300				
Gestation Age (w)	0.929	0.102	0.851	1.015	0.961	0.652	0.809	1.142
IMV (d)	1.005	0.306	0.995	1.016	0.990	0.315	0.969	1.010
Supplementary O2 (d)	1.004	0.096	0.999	1.008	1.002	0.698	0.993	1.010
CPAP (d)	1.009	0.277	0.993	1.024	1.001	0.928	0.980	1.022
Surfactant doses (n)	1.173	0.190	0.924	1.489	1.079	0.572	0.829	1.405
Post-natal steroids (y/n)	1.443	0.121	0.908	2.294	1.482	0.192	0.821	2.677
Sepsis (n)	1.453	0.104	0.926	2.278	1.359	0.241	0.814	2.270
Respiratory admission (y/n)	1.182	0.438	0.775	1.804	1.145	0.557	0.728	1.801
**Peribronchial thickening**								
Antenatal Steroids (y/n)	1.235	0.640	0.510	2.988				
Birth weight z-score	1.160	0.410	0.815	1.651				
Gestation Age (w)	1.069	0.323	0.936	1.221	1.275	0.176	0.897	1.813
IMV (d)	1.002	0.807	0.987	1.017	0.978	0.163	0.949	1.009
Supplementary O2 (d)	1.005	0.151	0.998	1.011	1.015	**0.030***	1.001	1.028
CPAP (d)	0.928	**0.002***	0.886	0.973	0.902	**0.005***	0.838	0.970
Surfactant doses (n)	1.111	0.571	0.773	1.596	1.322	0.230	0.838	2.084
Post-natal steroids (y/n)	1.858	0.072	0.947	3.646	3.144	**0.043***	1.038	9.521
Sepsis (n)	0.967	0.926	0.480	1.948	0.970	0.952	0.358	2.628
Respiratory admission (y/n)	4.009	**<0.001***	1.890	8.505	4.054	**0.003***	1.597	10.290

**Total Score**								
Antenatal Steroids (y/n)	1.349	0.252	0.808	2.254				
Birth weight z-score	1.116	0.321	0.899	1.386				
Gestation Age (w)	0.908	**0.024***	0.835	0.987	0.976	0.769	0.829	1.149
IMV (d)	1.008	0.118	0.998	1.018	0.989	0.238	0.971	1.007
Supplementary O2 (d)	1.006	**0.006***	1.002	1.010	1.005	0.179	0.998	1.013
CPAP (d)	1.007	0.362	0.992	1.021	0.996	0.695	0.977	1.016
Surfactant doses (n)	1.281	**0.027***	1.028	1.596	1.194	0.152	0.937	1.521
Post-natal steroids (y/n)	1.440	0.094	0.940	2.206	1.229	0.467	0.705	2.144
Sepsis (n)	1.478	0.064	0.977	2.235	1.240	0.368	0.776	1.980
Respiratory admission (y/n)	1.391	0.094	0.946	2.047	1.262	0.270	0.835	1.908

Table E17. Neonatal factors as risks ratios for lung structure abnormalities in preterm adolescents and young adults. *$p<0.05$.
